# Enhancing the anticancer immune response with the assistance of drug repurposing and delivery systems

**DOI:** 10.1002/ctm2.1320

**Published:** 2023-07-05

**Authors:** Zile Fu, Xiaoming Zhang, Yanfeng Gao, Jia Fan, Qiang Gao

**Affiliations:** ^1^ Department of Liver Surgery and Transplantation and Key Laboratory of Carcinogenesis and Cancer Invasion (Ministry of Education) Liver Cancer Institute Zhongshan Hospital Fudan University Shanghai China; ^2^ The Center for Microbes Development and Health Key Laboratory of Molecular Virology and Immunology Chinese Academy of Sciences Shanghai China; ^3^ School of Pharmaceutical Sciences (Shenzhen) Sun Yat‐sen University Shenzhen China; ^4^ Key Laboratory of Medical Epigenetics and Metabolism Institutes of Biomedical Sciences Fudan University Shanghai China; ^5^ Human Phenome Institute Fudan University Shanghai China

**Keywords:** anticancer immune response, drug repurposing, drug delivery system, immunotherapy

## Abstract

**Background:**

The immune system plays a pivotal role in the initiation, evolution, invasion and metastasis of cancer. Therapeutics aiming at modulating or boosting anticancer immune responses have experienced immense advances during the past decades, for example, anti‐PD‐1/PD‐L1 monoclonal antibodies.

**Main body:**

Concomitant with advancements in the understanding of novel mechanisms of action, conventional or emerging drugs bearing the potential to be repurposed for enhancing anticancer immunity have been identified. Meanwhile, ongoing advances in drug delivery systems enable us to utilise novel therapeutic strategies and impart drugs with fresh modes of action in tumour immunology.

**Conclusion:**

Herein, we systemically review these kinds of drugs and delivery systems that can unleash the anticancer response through various aspects, including immune recognition, activation, infiltration and tumour killing. We also discuss the current caveats and future directions of these emerging strategies.

## Graphical abstract

Repurposed drugs and drug delivery systems could help overcome immunosuppression induced by cancer.

Drugs unveiled to have immunostimulatory effects in cancer treatment span multiple categories.

Well‐designed structures and rational cargos enable delivery systems to stimulate the anticancer immune response.

The utilisation of repurposed drugs and delivery systems presents a promising avenue for enhancing cancer immunotherapy.

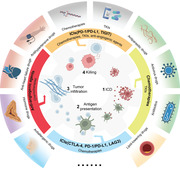

## 1 INTRODUCTION

There exist highly complex interactions between the immune system and cancer.^1^ Accumulations of genetic, epigenetic and phenotypic abnormalities during tumourigenesis and progression undergo immunosurveillance by various cell types in the innate and adaptive immune system, including macrophages, natural killer (NK) cells, CD8^+^ T cells, tissue‐resident memory T cells and so forth.^2^ Meanwhile, avoiding immune destruction is deemed a hallmark of cancer.^3^ Well‐studied phenomena, such as exhaustion of T cells, M2 polarisation of macrophages and recruitment of myeloid‐derived suppressor cells (MDSCs), underscore the capability of cancer to shape an immunosuppressive tumour microenvironment (TME).^4^ The strong association between cancer and the immune system has brought forth the prosperity of tactic developments for anticancer immune responses.

**FIGURE 1:**
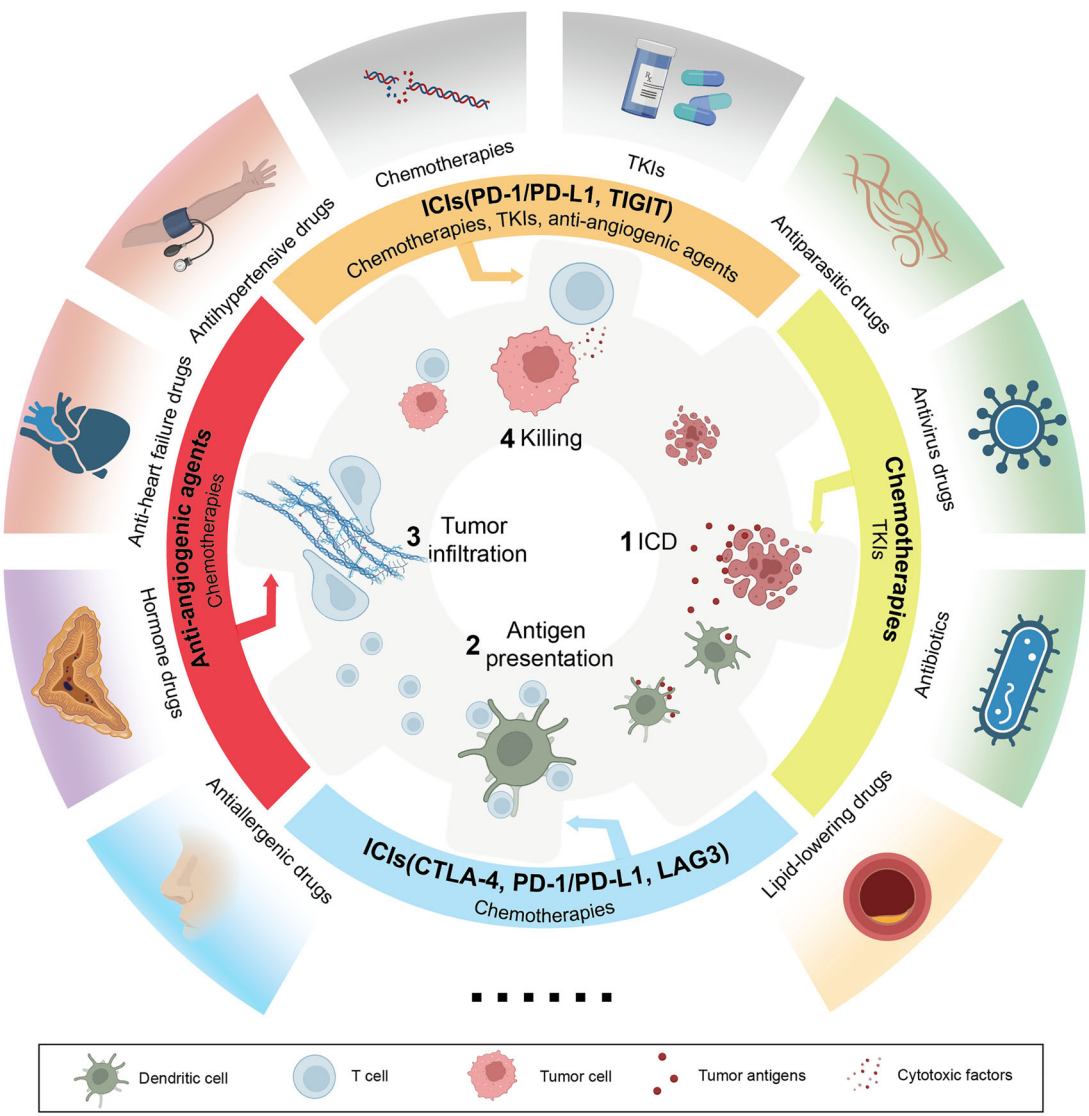
Schematic showing the ‘chain reaction’ of the anticancer immune response and related anticancer therapeutics. Inner circle: once immunogenic cell death (ICD) happens, adequate tumour antigens are released into the tumour microenvironment (TME). Dendritic cells (DCs) can uptake and process tumour antigens. Processed antigens will be presented on their cell membrane in the form of peptide‐major histocompatibility complex (pMHC) to activate T cells via T cell receptor (TCR)‐pMHC interaction, and several co‐stimulatory signals (e.g., CD80/86) will facilitate their priming into effector T cells (T_eff_). After being primed, T_eff_ cells will traffic in blood vessels and infiltrate into tumour sites. After recognising tumour cells through TCR‐pMHC interaction, T_eff_ cells can kill tumour cells through cytotoxic factors (e.g., perforin and granzyme B). Middle circle: current anticancer therapeutics that could push the ‘gear’ forward in certain steps of anticancer immune responses. Outside circle: repurposed drugs holding the potential as immunostimulators for combination with current anticancer therapeutics to promote anticancer immune response in different steps.

Immunotherapy represents an essential approach in the current landscape of cancer treatment. Clinically approved immunotherapeutics mainly include immune checkpoint inhibitors (ICIs; e.g., PD‐1/PD‐L1, CTLA‐4 and lymphoid activation gene 3 [LAG‐3] monoclonal antibodies [mAbs]), cytokine therapies (e.g., interleukin–2 [IL‐2] and interleukin–12 [IL‐12]) and adoptive cell transfer (e.g., chimeric antigen receptor T [CAR‐T] cells).^5^, ^6^ Development of cancer immunotherapy mainly depends on the mechanisms of the ‘chain reaction’ in anticancer immune response (Figure 1). However, not all patients have ideal therapeutic responses to these treatments, and the high resistance rate urges rational new treatment strategies.^7^ Multifarious combination strategies, including chemotherapeutics with ICIs, ICIs targeting multiple immune checkpoints, tyrosine kinase inhibitors (TKIs) with ICIs and anti‐angiogenics with ICIs, have been thus recommended as the first‐line treatments in various types of cancer to tackle these obstacles (Figure 1).^8^, ^9^, ^10^

During the pursuit of overcoming immunosuppression, alternative approaches have been explored, among which drug repurposing and drug delivery systems (DDSs) have emerged as encouraging strategies. Repurposing existing drugs that were developed for other indications towards new uses is a promising pipeline in drug development (Figure 1). Several advantages of drug repurposing over *de novo* drug development have been acknowledged. One is that repurposed drugs have already been tested for their safety and toxicity, and passed through necessary dose explorations, which vastly reduces approval times for their new applications. Also, their structures, pharmacokinetics, pharmacodynamics or even mechanisms of action are vastly decoded.^11^ However, it is important to mention that before any drug can be repurposed, it must undergo rigorous and additional preclinical and clinical research, regardless of whether the drug has previously undergone toxicity testing and dose exploration. In recent years, due to the exponential data volume in oncological research (e.g., next‐generation sequencing and proteomics data), data‐driven searching for drug repurposing is gradually taking the lead in strategy selection and has generated large‐scale drug response repositories such as PRISM (https://depmap.org/repurposing/) and LINCS (https://lincsportal.ccs.miami.edu/signatures/home) that can facilitate the research community for direct drug repurposing mining.^12^, ^13^

The majority of anticancer drugs harbour off‐target effects, which may dampen their tumour‐killing efficacies and are associated with some major or occasionally fatal complications.^14^ To overcome these drawbacks in systemic administrations of anticancer drugs, many DDSs have been constructed to enhance the spatiotemporal targeting ability. Building on the improvements of biomaterials regarding permeability and retention in tumours, various DDSs like liposomes, polymers, mesoporous silica nanoparticles, metal‐organic frameworks and carrier‐free DDSs (e.g., self‐assembled drugs and amphiphilic drug‐chemogene conjugates) are acquiring prominence in carrying anticancer agents for their outperforming efficacy, compared to plain drugs tested on preclinical models and in clinical trials.^15^, ^16^, ^17^, ^18^, ^19^ Simultaneously, pharmacokinetic meliorations have been achieved through surface modifications, rational camouflages (e.g., cancer cell membrane camouflage) or multistage release, which can further improve the performance of DDSs.^20^, ^21^, ^22^

In this review, we highlight drugs harbouring the potential to be repurposed and DDSs that can exert a positive influence on the anticancer immune response. The underlying mechanisms are discussed according to the steps of the tumour immune ‘chain reaction’. In addition, we propose the future practice of repurposed drugs and DDSs regarding the immunological basis, tumour heterogeneity and potential combinations of repurposed drugs and DDSs with immunotherapy.

## 2 ANTIGEN GENERATION, PRESENTATION, RELEASE AND CAPTURE

The cancer cell‐specific killing predominantly depends on enough quality and quantity of primed CD8^+^ T cells. Tumour antigens with ample immunogenicity should be untaken by antigen‐presentation cells (APCs), mainly dendritic cells (DCs), through endocytosis and then processed and presented to prime naïve T cells. Only then can CD8^+^ T cells be activated and exert cytotoxic effects by recognising peptide‐major histocompatibility complex (pMHC) displayed on the cancer cell membrane. Nevertheless, cancer cells are capable of dying in non‐immunogenic forms and dampening antigen uptake by APCs.^23^ Meanwhile, suppression of cancer cells’ own antigen presentation via downregulation of MHC molecules or other defects in antigen presentation machinery (APM) can hamper the killing effects of cytotoxic T lymphocytes (CTL). Repurposed drugs (Table 1) and novel DDSs (Table 2) have been oriented to induce immunogenic cell death (ICD), promote antigen release and presentation of cancer cells or directly augment the function of APCs.

**TABLE 1:** Drugs harbouring the potential to be repurposed for enhancing anticancer immune response.

Process	Drug name	Major indication	Mechanism of actions	Ref.
Antigen generation, presentation, release and capture				
Immunogenic cell death (ICD) for effective antigen release	DOX	Several malignancies	Cascade‐mediated cell death	^24^
	Statins (e.g., simvastatin)	Hyperlipidemia	Inducing ER stress and CRT expression	^200^
	Lomitapide	Familial hypercholesterolemia	Suppressing mTOR signalling thus inducing autophagic cell death	^201^
	Digoxin	Heart failure	Activation of ERp57 and disorder of the intracellular ion	^202^
	Dihydroartemisinin	Malaria	Elevating intracellular ROS level	^203^
Cancer cell antigen generation and presentation	Olaparib	Several malignancies	Amplifying the genome instability through inhibiting PARP1 and PARP2 and triggering neoantigen generation	^204^
	LB100	Several malignancies	Amplifying the genome instability through inhibiting PP2A and triggering neoantigen generation	^45^
	Temozolomide	Glioblastoma	Inducing hypermethylation of *MGMT* promotor regions thus impairing DNA mismatch repair	^46^
	Entinostat	Several malignancies	Boosting the generation of cancer neoantigens through inhibiting HDAC1	^205^
	Abemaciclib	Several malignancies	Increasing the expression of major histocompatibility complex (MHC) molecules	^206^
	Albendazole	Intestinal parasites	Increasing the expression of p53 and arresting cell cycle at G2/M phase	^207^
	Indisulam	Lung cancer	Increasing neoantigens through inducing splicing errors	^208^
	Domatinostat	Several malignancies	Inhibiting HDAC1 to restore the expression of antigen presentation machinery components	^59^
	Statins (e.g., simvastatin)	Hyperlipidemia	Upregulation of tumour cell MHC	^200^
	Aspirin	Pain, fever and inflammation	Restricting the secretion of cytokines disrupting cancer cell antigen presentation such as IL‐4 and IL‐10	^209^
Infiltration and function of antigen‐presentation cells (APCs)	Seasonal flu vaccine	Seasonal flu	Recruiting dendritic cells (DCs) to tumour sites	^210^
	Statins (e.g., lovastatin)	Hyperlipidemia	Increasing the number of tumour‐infiltrated DCs	^200^
Anticancer immune response outside the tumour				
Activation and proliferation of immune effector cells	Decitabine	Myelodysplastic syndrome	Reducing the AIRE expression in medullary epithelial cells to break the central tolerance	^211^
	Propranolol	Hypertension	Inhibition of the expansion and function of both circulating and intratumoural myeloid‐derived suppressor cells (MDSCs) through the blockade of β‐adrenergic signalling	^85^
	rhTSH	Differentiated thyroid carcinoma	Increasing the circulating natural killer (NK) cell number	^87^
	Atovaquone	Protozoa infection	Inhibition of the differentiation into T_reg_ cells through suppressing the complex‐I‐driven bio‐oxidation	^212^
	Ibrutinib	Mantle cell lymphoma	Increasing the circulating CD4^+^ and CD8^+^ T cells through inhibition of inducible T cell kinase signalling in T cells	^87^
	Azelnidipine	Hypertension	Increasing the CD8^+^ T cells in tumour‐draining lymph nodes and spleen through dual blockade of SIRPα and PVR to suppress CD47/SIRPα and TIGIT/PVR signalling	^213^
	Amlexanox	Recurrent aphthous ulcer	Enhancing the IFN‐γ production of DCs	^214^
	Ritonavir	HIV infection	Increasing the circulating CD4^+^ T cells through inhibiting the HSP90‐mediated apoptosis	^215^
	Abemaciclib	Several malignancies	T cell activation and proliferation through NFAT signalling	^206^
Sustainable immune response against tumour (re)challenge	Dacarbazine	Malignant melanoma	Boosting the T_em_ cells	^216^
			Enlarging the TCR repository	^94^
Infiltration of effector cells				
Effector cell‐recruiting cytokine signals	Seasonal flu vaccine	Seasonal flu	Promoting the tumour infiltration of CD8^+^ T cells through upregulating the expression of CCL5 and many other chemokines	^210^
	Abemaciclib	Several malignancies	Promoting the tumour infiltration of CD8^+^ T cells and T helper 1 cells through upregulating the release of CXCL10 from tumour cells	^217^
	Chloroquine	Malaria	Promoting the tumour infiltration of NK cells through activating the TLR3/IFN‐β/RIG‐1/CCL3 axis of tumour cells	^218^, ^219^
	Indomethacin	Pain, fever and inflammation	Upregulating the release of immune effector cell‐recruiting cytokines such as CXCL9 and CXCL10 from cancer cells	^220^
Extracellular matrix (ECM) modulation	Tranilast	Allergy	Inhibiting the TGF‐β signalling of cancer‐associated fibroblasts (CAFs) to reduce the collagen synthesis	^221^, ^222^
	Macitentan	Pulmonary arterial hypertension	Inhibiting the endothelin‐mediated activation of cancer–associated fibroblasts (CAFs)	^123^
	Metformin	Type 2 diabetes mellitus	Inhibiting angiogenesis through impairing the function of precursor endothelial cells	^223^
Enhancement of the immune killing effects				
Alternatives for immune checkpoint inhibitor (ICI) besides monoclonal antibodies (mAbs)	Cefepime	Infection	Downregulating PD‐L1 expression through inducing ubiquitination	^224^
	Copper chelators (e.g., Penicillamine)	Wilson's disease	Inhibiting the phosphorylation of STAT3; promoting the ubiquitination and degradation of PD‐L1	^225^
	Macitentan	Pulmonary arterial hypertension	Downregulating PD‐L1 exosome secretion from tumour cells	^123^
	Metformin	Type 2 diabetes mellitus	Downregulating PD‐L1 expression through mediating its ER‐associated degradation	^226^
Stimulation of tumour‐infiltrated effector cells	Propranolol	Hypertension	Enhancing the function of infiltrated CD8^+^ T cells through intensifying glycolysis in T cells	^227^
	Vitamin C	Vitamin C deficiency	Inducing the *KIR* promoter demethylation through enhancing the catalytic efficiency of TET	^228^
	Aspirin	Pain, fever and inflammation	Enhancing the function of infiltrated CD8^+^ T cells through inhibiting the activation of platelets in tumour microenvironment (TME)	^229^
	Metformin	Type 2 diabetes mellitus	Enhancing the function of infiltrated CD8^+^ T cells through activating several metabolic programs including fatty acid oxidation and glycolysis	^230^
Suppression of immunosuppressive cells in TME	Tazemetostat	Epithelioid sarcoma	Promoting the M1 polarisation of tumour‐associated macrophages (TAMs) through downregulating the methylation level of H3K27 at STAT3 promotor regions	^157^
	MAO inhibitor (e.g., phenelzine)	Depression	Constraining the M2 polarisation of TAMs through reducing ROS levels and the activation of JAK‐STAT6 signalling	^158^
	Chloroquine	Malaria	Promoting the repolarisation of TAMs to M1‐like phenotype	^231^
	Maraviroc	CCR5‐tropic HIV infection	Preventing the polarisation of macrophages to TAMs	^113^
	Tranilast	Allergy	Promoting the M1 polarisation of TAMs through inhibiting the TGF‐β signalling of TAMs	^221^, ^222^
	Imipramine	Depression	Inhibiting H1 histamine receptor and driving the M1 polarisation of TAMs	^232^
	Fexofenadine	Allergic rhinitis	Inhibiting the membrane location of VISTA on TAMs	^233^
	Zoledronic acid	Osteoporosis caused by menopause or malignancies	Promoting the M1 polarisation of TAMs through activating the TLR4/NF‐κB pathway	^162^, ^234^
	Aspirin	Pain, fever and inflammation	Promoting the repolarisation of TAMs to M1‐like phenotype	^235^
	Atovaquone	Protozoa infection	Reducing intratumoural T_reg_ cells and MDSCs	^236^
	Captopril	Hypertension	NA	^237^
	Imatinib	Leukaemia or other malignancies	Inhibition of T_reg_ functions through inhibiting STAT3 and STAT5 phosphorylation	^168^
	Ibrutinib	Mantle cell lymphoma	Suppressing the MDSC function	^238^
	Fulvestrant	Hormone receptor‐positive breast cancer	Inhibition of MDSC functions	^239^, ^240^
Cancer immunometabolism in TME	DCA	Insulin resistance	Reducing lactate in TME through inhibiting PDK in mitochondria	^173^
	Carbidopa	Parkinson disease	Activating AhR to suppress the constitutively and IFN‐γ‐induced expression of IDO1	^241^
	Eflornithine	African trypanosomiasis	Suppressing the activation of ODC to reduce the immunosuppressive polyamine levels in TME	^180^
	Ceritinib	Non‐small cell lung cancer	Decreasing adenosine generation through direct inhibition of CD39	^242^
	Metformin	Type 2 diabetes mellitus	Suppressing the CD39/CD73 metabolic axis through inhibition HIF‐1α after AMPK activation	^243^
	Stannsoporfin	Hyperbilirubinemia	Reducing CO in TME through inhibiting HO‐1 in TAMs	^244^
	Statins (e.g., Simvastatin)	Hyperlipidemia	Reversing the CD8+T cell exhaustion through reducing cholesterol in TME	^245^
Tumour inflammatory microenvironment	Celecoxib	Arthritis	Reducing the PD‐L1 level in TME	^183^
	Tocilizumab	Rheumatoid arthritis	Increasing the number of effector CD4^+^ and CD8^+^ T cells in TME through blocking IL‐6 signalling	^246^

Abbreviations: AhR, aryl hydrocarbon receptor; AIRE, autoimmune regulator; CRT, calreticulin; CO, carbon monoxide; DCA, dichloroacetate; DOX, doxorubicin; ER, endoplasmic reticulum; HSP, heat shock protein; H3K27, histone H3 at lysine 27; HO‐1, heme oxygenase–1; MAO, monoamine oxidase A; NA, not applicable; NFAT, nuclear factor of activated T cells; ODC, ornithine decarboxylase; PVR, poliovirus receptor; ROS, reactive oxygen species; rhTSH, recombinant human thyroid‐stimulating hormone; SIRPα, signal regulatory protein alpha; TLR, Toll–like receptor.

**TABLE 2:** Examples of drug delivery systems for enhancing anticancer immune response.

Effects	Carrier	Agent	Modification/sensitivity	Mechanism of action	Ref.
Antigen generation, presentation, release and capture					
ICD for effective antigen release	PMO	DOX	TRAIL	Enhancing the ICD‐inducing capability of DOX	^27^
	Antibody	Anthracyclines	NA	Enhancing the ICD‐inducing capability of anthracyclines	^247^
	Liposome	DOX + disulfiram	NA	Disulfiram elevates the cellular ROS level, induces apoptosis and orchestrates the ICD‐inducing capability of DOX	^248^
	TiO_2_ NP	NA	Calcium phosphate	Degrading in the acidic TME and inducing ICD through elevated ROS and Ca^2+^ levels	^249^
Infiltration and function of APCs	MSN	CpG + PS	PEGylation + hypoxia‐responsive linker	Promoting the activation and maturation of DCs	^250^
Infiltration of effector cells					
Effector cell‐recruiting cytokine signals	Lipid NP	siADORA2A	Anti‐CD45RO antibody	Knockdown of A_2A_R to block the adenosine‐mediated immunosuppressive effects on memory T cells	^251^
ECM modulation	PEG‐poly(lactic‐co‐glycolic acid) (PLGA) NP	Macitentan	NA	Inhibiting the endothelin‐mediated activation of CAFs	^123^
	Exosome‐like nanovesicles	FAP	Tumour antigens	Inducing the specific T cell response against FAP^+^CAFs	^119^
	OHC‐PEG‐CHO/PEI/PLG + PLG‐*g*‐mPEG	shPD‐L1 + *pSpam1* + DOX	NA	Inducing ICD; degrading ECM hyaluronic acid; knockdown of PD‐L1 expression in tumour cells	^120^
	hypervesiculating *Escherichia coli*	Plasmid encoding cytolysin A and Haase	NA	Mediating the degradation of hyaluronic acid and lysis of tumour cells	^122^
	Human recombinant PH20 hyaluronidase		PEGylation	Digesting the hyaluronic acid in TME	^252^
	Ace‐DEX NP	Collagenase	NA	Digesting collagen in tumour ECM	^253^
Tumour vasculature normalisation	Empty gold NP	Folic acid		Heparin‐binding of growth factors; inhibition of Smad2/3 signalling in tumour endothelial cells	^254^
	N3‐PEG‐p*Lys*‐p*Phe* amphiphile polypeptide	Probe X + DOX	Linked cRGD	Relieving the tumour angiogenesis	^134^
	Lipid‐PLGA NP	DNIC	PEGylation	Increasing the tumour vessel perfusion, resulting in elevated infiltration of T cells	^255^
	Carbon nanotubes	siVEGF + candesartan	PEI	Inhibiting the tumour angiogenesis through blocking AT_1_R and VEGF/VEGFR signalling	^256^
Enhancement of the immune killing effects					
Alternatives for ICI besides mAbs	Fe_3_O_4_ NPs	siPD‐L1	Microglial membrane	Targeted inhibition of PD‐L1 at mRNA level in tumour cells	^144^
	*E. coli* Nissle	Gene encoding Anti‐PD‐L1 and Anti‐CTLA‐4 nanobody	NA	Intratumourally expressing Anti‐PD‐L1 and Anti‐CTLA‐4 nanobodies	^188^
Suppression of immunosuppressive cells in TME	liposome	Zoledronic acid	SA	Killing M2‐like TAMs and reversing their phenotype to M1 macrophages	^234^
	DSPE‐PEG and PLGA‐hyd‐PEG	Chloroquine + platinum + ^D^PPA peptide	Ultrasonic and pH dual responsiveness	Repolarisation of TAMs to M1 phenotypes; Blockade of the PD‐L1 in tumour cells	^231^
	Liposome	Zoledronic acid + MnO_2_ + PS	LyP‐1 peptide + PEGylation	Killing M2‐like TAMs and reverse their phenotype to M1 macrophages; photodynamic effects on tumour cells	^163^
	haemoglobin–poly(ε‐caprolactone) conjugate	DOX + O_2_	NA	Killing M2 TAMs through DOX and reducing their recruitment through oxygen release	^257^
	*E.coli*	R848 + DOX	PLGA‐R848 complex + PLGA‐DOX complex	Repolarisation of M2 TAMs to M1 phenotypes	^258^
	mPEG‐block‐poly(l‐alanine) hydrogel	Regorafenib + LY3200882	NA	Reducing the number of MDSCs and TAMs in TME; polarising M2 TAM to M1 phenotypes; inducing ICD	^169^
Cancer immunometabolism in TME	PEG‐PLGA NP	DCA	Terminal triphenyl phosphonium linked to PEG	Reducing lactate level in TME through inhibiting PDK	^173^
	Self‐assembled drug NP (PS + NLG919 + OXA)	PEGylation		Reducing kynurenine level in TME through inhibiting IDO1; direct killing effects on tumour cells; inducing ICD	^176^
	mPEG‐poly(L‐phenylalanine‐co‐L‐cystine) nanogel	DOX + 1MT	NA	Reducing kynurenine level in TME through inhibiting IDOs; inducing ICD	^259^

Abbreviations: 1MT, 1‐methyl‐DL‐tryptophan; A_2A_R, receptor A_2A_; Ace‐DEX, acetylated dextran; ADORA2A, adenosine receptor A_2A_; AT_1_R: angiotensin II type‐1 (AT_1_) receptor; CHO, glycol; cRGD, cyclic RGD peptide; DCA, dichloroacetate; DNIC, dinitrosyl iron complex; DSPE, 1,2‐distearoyl‐sn‐glycero‐3‐phosphoethanolamine; hyd, hydrazone bonds; MnO_2_, manganese dioxide; MSN, mesoporous silica nanoparticles; NA, not applicable, NG, nanogel; NP, nanoparticle; OHC, aldehyde‐modified; OXA, oxaliplatin; PEG, polyethylene glycol; PEI, polyethyleneimine; PLG, poly(L‐glutamic acid); PMO, periodic mesoporous organosilica; SA, sialic acid; TRAIL, tumour necrosis factor‐related apoptosis‐inducing ligand.

### 2.1 ICD for effective antigen release

The boundary between ICD and other forms of cell death including ferroptosis, apoptosis and pyroptosis is not clear. ICD relies on the emission and detection of damage–associated molecular patterns (DAMPs including the release of adenosine triphosphate (ATP), membrane location of endoplasmic reticulum chaperones, IL‐1β production and so forth.^23^ The immunomodulating mechanisms of traditional chemotherapeutics were largely neglected in clinical application. Nevertheless, part of these chemotherapeutics was unveiled to function through immune‐related mechanisms, including the induction of ICD.^24^ Despite that the combination of chemotherapeutics with ICIs is approved for the treatment of several cancer types (e.g., pembrolizumab plus platinum drugs in the treatment of non‐small cell lung cancer),^25^ the immunogenicity of cancer cell death mediated by these drugs was considered insufficient, and chemotherapy‐related immunosuppression (e.g., more immunosuppressive chemokines secreted by cancer cells) together with the whole‐body toxicity could hamper their therapeutic efficacy or even be associated with negative results of clinical trials.^26^

To overcome these drawbacks, DDSs have been harnessed to enhance the ICD‐inducing ability of cytotoxic agents through prolonging drug circulation time, increasing drug uptake by cancer cells or other pro‐ICD modifications in DDS designation. Tumour necrosis factor‐related apoptosis‐inducing ligand (TRAIL)‐decorated nanoparticle was constructed to deliver doxorubicin in a murine breast cancer model.^27^ It was demonstrated that TRAIL‐modified DDS promoted doxorubicin on its ICD‐inducing capability, leading to drug accumulation in tumours and activation of the apoptosis pathway. An acid‐sensitive polyethylene glycol (PEG)‐decorated calcium carbonate nanoparticle loaded with curcumin, an inducer of mitochondrial Ca2+ overloading, was synthesised and assessed for its ICD‐inducing capability. Enhanced ICD triggered by accumulation of reactive oxygen species (ROS) was confirmed and can be further augmented by ultrasound stimulation.^28^ Antibody–drug conjugate (ADC) is another kind of DDS for delivering cytotoxic agents with tumour specificity, and several clinical trials have proved ADCs as a powerful agent in cancer treatment, leading to their clinical application (e.g., Trodelvy in the treatment of advanced breast cancer).^29^, ^30^ Not surprisingly, ADCs had enhanced ICD‐inducing capacity, compared with plain chemotherapeutics, and the combinations with anti‐PD‐1 therapy have advanced to clinical trials.^31^ This is in accordance with the observation that cancer cell lysates could serve as immune adjuvants, indicating that increased cancer cell death under conventional chemotherapeutics is more likely to induce the anticancer immune response.^32^ Thus, targeted delivery and improved pharmacokinetics brought by DDSs might provide a novel arena for cancer chemotherapeutics regarding immunomodulatory aptitudes.

In addition to chemotherapeutics, an increasing number of drugs demonstrating ICD‐inducing ability have been identified. As a paradigmatic example, statins were initially developed to inhibit the key enzyme in cholesterol biosynthesis, 3‐hydroxy‐3‐methylglutaryl coenzyme A, to ameliorate hyperlipidemia. Retrospective analyses have revealed a reduced risk of death among statin users with cancer.^33^ Mechanistically, statin can increase the eukaryotic initiation factor 2 alpha (eIF2α) phosphorylation and trigger the translocation of calreticulin to the cancer cell membrane, resulting in ICD.^34^ Based on these findings, interventional clinical studies including the combination with traditional chemotherapeutics were carried out to assess the possible anticancer effects of statins.^35^ Cancer stem cells (CSCs) represent a distinct cell population in cancer, and their advantages of evading immunosurveillance can directly lead to cancer recurrence.^36^ Exposing CSCs to immune detection and clearance is an important concept in cancer treatment. The anti‐alcoholism agent, disulfiram, was portrayed to induce ICD of breast CSCs through upregulating ROS levels and activating the inositol requiring enzyme 1 alpha (IRE1α)/ X‐box‐binding protein 1 (XBP1) axis.^37^ This success in preclinical models might pave the way for CSC‐based immunotherapies.

### 2.2 Cancer cell antigen generation and presentation

Another impeding factor in generating potent CD8^+^ T cell‐mediated killing is attenuated antigen processing and presentation in cancer cells.^38^ To guarantee the recognition by T_eff_ cells, possible solutions to restore antigen presentation on cancer cells have been explored.

#### 2.2.1 Generation of tumour antigens or neoantigens

**FIGURE 2:**
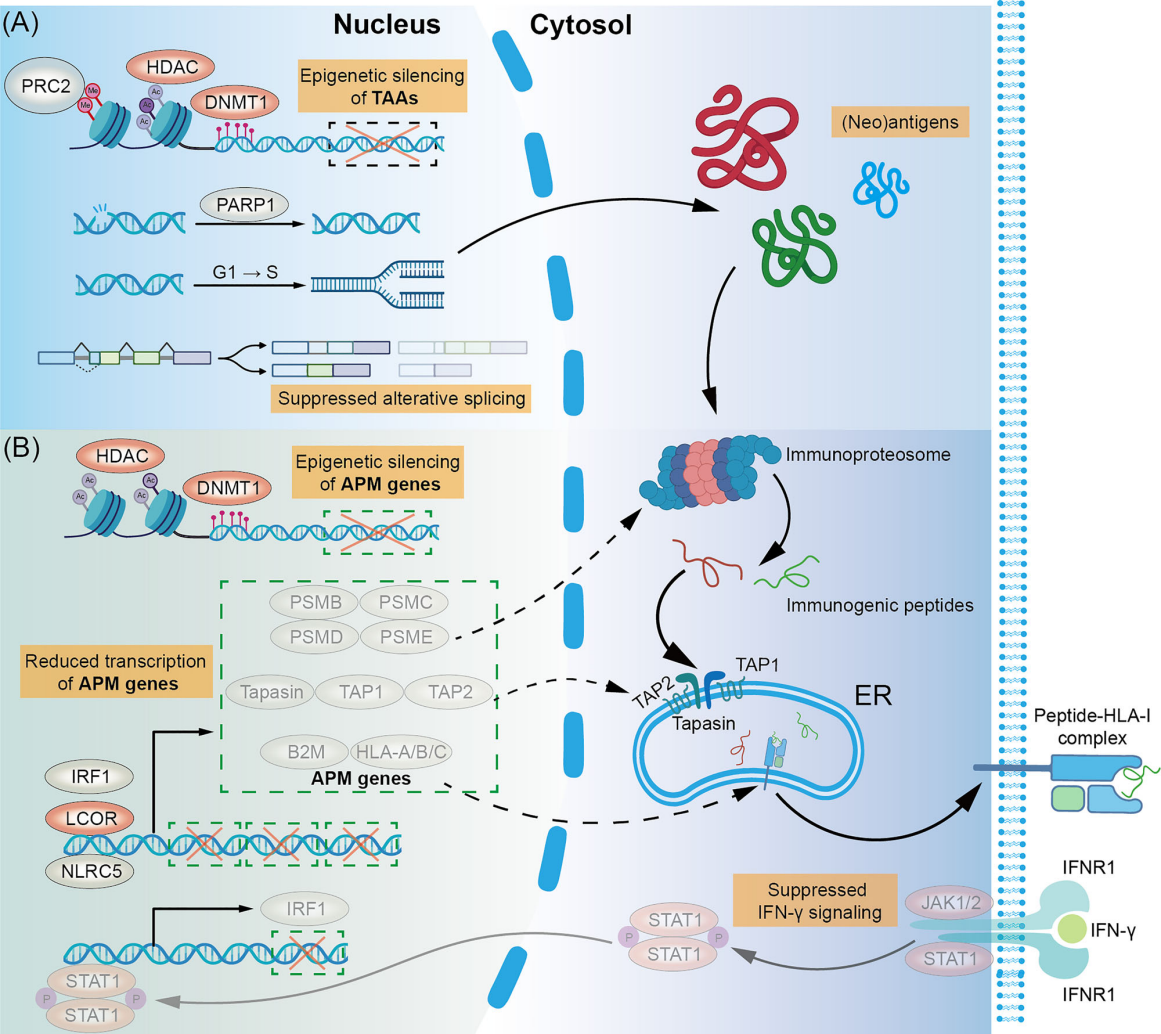
Underlying mechanisms of reduced antigen generation and suppressed antigen presentation in cancer cells. (A) Various mechanisms are involved in the loss‐of‐immunogenicity of cancer cells, and those targetable for repurposed drugs and drug delivery systems (DDSs) are summarised here. Epigenetic silencing of gene regions encoding tumour antigens can be induced by promotor hypermethylation, histone methylation or histone deacetylation by epigenetic modifying enzymes involving HDACs, DNMT1 and complex such as PRC2. Meanwhile, intact DNA damage response guaranteed by PARPs (mainly PARP1), unencumbered cell cycle progression and suppressed premature mRNA alternative splicing could also reduce the generation of immunogenic endogenous proteins. (B) Antigen presentation machinery (APM) deficiency is another cause of low immunogenicity of cancer cells. Epigenetic silencing can also occur on APM genes. Meanwhile, diminished functions of crucial transcriptional factors including IRF1, ligand‐dependent corepressor (LCOR) and NLRC5 can affect protein levels of immunoproteasome subunits (PSMB, PSMC, PSMD, PSME, etc.), peptide transporters (TAP1, TAP2, Tapasin, etc.) and human leukocyte antigen (HLA)‐I subunits (HLA‐A/B/C and B2M). The master regulator of APM, IFN‐γ signalling, can also be impeded through downregulating key signal transduction molecules including JAK1/2 and STAT1. The red background represents targetable molecules or pathways in terms of elevating the antigen generation and presentation levels in cancer cells. The translucent elements represent decreased abundances of proteins due to the impaired APM. The translucent arrows represent suppressed signal transduction. ER, endoplasmic reticulum; TAA, tumour‐associated antigen.

Owing to the widespread genetic and epigenetic abnormalities, cancer cells are capable of re‐expressing certain germ cell proteins (cancer‐testis antigens) and generating proteins that are completely absent from normal tissues (neoantigens; Figure 2).^39^ Crucial mutational events comprising DNA damage response (DDR) deficiency and microsatellite instability can enrich neoantigen generation in cancer cells and have been associated with better response to immunotherapy.^40^ However, the dampened surface presentation and immune clearance‐derived clonal evolution can consequently sequester their immunogenicity from evoking effective immune killing.^41^ In several clinical trials, personalised neoantigen vaccines have been developed and validated to generate tumour‐specific T cell responses.^42^ Various methods were also proposed targeting the endogenous generation of tumour (neo)antigens for conquering these obstacles.

‘Genome instability and mutation’ is known as a hallmark of cancer.^3^ Several mechanisms including DDR deficiency, such as polymerase epsilon and delta (*POLE* and *POLD1*) mutations and breast cancer susceptibility gene (*BRCA*) mutations, can contribute to increased genomic instability and mutability, thus resulting in an amplified antigenicity.^43^, ^44^ Cytotoxic drugs targeting the DDR process have been tested for their immunomodulatory effects beyond the capacity of triggering apoptosis. Among them, poly [ADP–ribose] polymerase (PARP) inhibitors can suppress critical proteins participating in DNA damage detection and mismatch repair to augment the neoantigen generation in cancer cells.^45^ Alkylating agents can impair the expression of DDR enzymes through promotor hypermethylation instead of direct inhibition.^46^ Immunogenic tumour antigens can be epigenetically muted in cancer cells to escape immune surveillance and killing.^47^ The altered histone modification and DNA methylation landscape led by inhibitors against histone‐modifying enzymes including DNA methyltransferase (DNMT) and HDAC were demonstrated to facilitate cancer (neo)antigen generation. Through applying decitabine to inhibit the function of DNMT in human lung cancer cell line, the cancer‐testis antigen *SPESP1* was re‐expressed and elicited T cell immune response in vitro.^48^ The combination of DNMT inhibitor and PARP inhibitor in several preclinical cancer models also observed a synergistic effect of inducing ICD, further indicating the importance of genetic and epigenetic modulation in cancer therapy.^49^

TKIs stand out in current cancer treatment, and the immunomodulating effects of TKIs have been extensively investigated. TKIs can enhance cancer cell antigen presentation via both interferon (IFN) signalling‐dependent or independent manners.^50^ In the latter manner, increased MHC‐I binding neoantigens were generated and loaded to MHC‐I molecules in cabozantinib‐treated human papillary thyroid carcinoma.^51^ This was possibly linked to the cell‐cycle arrest and supported by the evidence that the Food and Drug Administration (FDA)‐approved CDK4/6 inhibitor, abemaciclib, can also increase the neoantigen levels derived from the degradation of G1/S phase‐enriched proteins.^52^

The potential source of neoantigens is not limited to genetic and epigenetic alterations.^53^ Post‐transcriptional events such as abnormal RNA splicing and truncated protein translation can also induce the generation of neoantigens. Recently, amplifying these aberrant biological processes in cancer cells for enhanced neoantigen generation turned out to be achievable after an in‐depth understanding of other cell cycle‐interfering drugs (Table 1). The anticancer immune response brought by these drugs may provide the rationale for their combinations with current cancer immunotherapies (Table 3).

**TABLE 3:** Interventional clinical trials assessing repurposed drugs mentioned in this review in combination with current anticancer treatments.

Repurposed drugs	Indication	Intervention	Phase (status)	Ref. or identifier
Atorvastatin	Advanced breast cancer	Letrozole ± atorvastatin	II (recruiting)	NCT02958852
Statin	Advanced NSCLC	PD‐1/PD‐L1 inhibitors + statin	NA	NCT05636592
Disulfiram	Advanced NSCLC	cisplatin and vinorelbine ± disulfiram	IIb (completed)	^260^
Rovalpituzumab Tesirine	Extensive‐Stage small cell lung cancer	Nivolumab + Rovalpituzumab Tesirine ± ipilimumab	I/II (terminated)	NCT03026166
Digoxin	Metastatic breast cancer	Capecitabine + digoxin	II (terminated)	NCT01887288
Olaparib	Resectable urothelial bladder cancer	Durvalumab + olaparib	II (completed)	NCT03534492
LB100	Untreated extensive‐stage small cell lung cancer	Standard chemotherapy + atezolizumab + LB‐100	I (recruiting)	NCT04560972
Temozolomide	Advanced/Metastatic malignant melanoma	Toripalimab ± temozolomide	II (recruiting)	NCT04884997
Domatinostat	Muscle invasive urothelial cancer	Nivolumab ± ipilimumab ± domatinostat	I (active, not recruiting)	NCT04871594
Entinostat	Advanced TNBC	Atezolizumab ± entinostat	I/II (completed)	NCT02708680
Indisulam	Relapsed or refractory A	Idarubicin and cytarabine + indisulam	II (completed)	NCT01692197
Abemaciclib	Locally advanced or metastatic breast cancer	Fulvestrant ± abemaciclib	III (active, not recruiting)	^261^
Denosumab	Advanced NSCLC	Standard chemotherapy ± denosumab	III/IV (completed)	NCT02129699
Aspirin	MSI‐H/dMMR or high TMB colorectal cancer	BAT1306 (PD‐1 antibody) + aspirin	II (recruiting)	NCT03638297
	Cervical/Uterine cancer	Pembrolizumab + immune modulatory cocktail + aspirin	II (completed)	NCT03192059
Propranolol	Newly diagnosed breast cancer	Neoadjuvant chemotherapy + propranolol	II (completed)	NCT01847001
Atovaquone	Locally Advanced NSCLC	Standard chemotherapy + atovaquone	I (active, not recruiting)	NCT04648033
Ritonavir	Advanced solid malignant tumours	DS‐8201a (Trastuzumab deruxtecan) + Ritonavir	I (active, not recruiting)	NCT03383692
Plerixafor	Metastatic pancreatic cancer	Cemiplimab + plerixafor	II (recruiting)	NCT04177810
Macitentan	Recurrent glioblastoma	Temozolomide + macitentan	I (terminated)	^262^
Penicillamine	Newly diagnosed glioblastoma	Radiotherapy + penicillamine	II (completed)	^133^
Metformin	Solid tumour malignancies	Nivolumab/Pembrozilumab + Metformin	II (recruiting)	NCT04114136
	NSCLC with EGFR mutations	Gefitinib ± metformin	III (recruiting)	NCT05445791
Ganetespib	Stage IIIb or IV NSCLC	Docetaxel ± ganetespib	IIb/III (terminated)	NCT01348126
Tazemetostat	Several solid tumours	Durvalumab + tazemetostat	II (recruiting)	NCT04705818
Phenelzine sulfate	Metastatic breast cancer	Nanoparticle albumin‐bound paclitaxel + phenelzine	I (completed)	NCT03505528
Chloroquine	Advanced or metastatic breast cancer	Chemotherapy + chloroquine	II (completed)	NCT01446016
	Advanced solid tumours	Carboplatin/Gemcitabine ± chloroquine	I (completed)	NCT02071537
Maraviroc	Refractory microsatellite stable metastatic colorectal cancer	Pembrolizumab + maraviroc	I (completed)	^263^
Imipramine	Recurrent glioblastoma	Lomustine + imipramine	II (recruiting)	NCT04863950
Zoledronate	Neoadjuvant therapy of triple negative breast cancer	Anthracyclines/taxanes ± zoledronate and atorvastatin	II (recruiting)	NCT03358017
Captopril	Recurrent glioblastoma	Temozolomide + captopril	I/II (completed)	NCT02770378
Ibrutinib	Advanced, refractory colorectal cancers	Pembrolizumab + ibrutinib	II (completed)	^264^
Fulvestrant	Advanced NSCLC	Erlotinib ± fulvestrant	II (completed)	NCT00100854
Eflornithine	Localised prostate cancer	Bicalutamide + eflornithine	II (completed)	NCT00086736
Ciforadenant	Advanced renal cell carcinoma	Ipilimumab + nivolumab + ciforadenant	I/II (recruiting)	NCT05501054
Celecoxib	Early‐stage colon cancer	Nivolumab and ipilimumab ± celecoxib	II (recruiting)	^265^
	Resectable oral cavity squamous cell carcinoma	Erlotinib + celecoxib	II (active, not recruiting)	NCT02748707
Tocilizumab	Several solid tumours	Ipilimumab + nivolumab + tocilizumab	II (recruiting)	NCT04940299

Abbreviations: dMMR, deficiency mismatch repair; NA, not applicable; NSCLC, non‐small cell lung carcinoma; MSI‐H, microsatellite instability‐high; TMB, tumour mutation burden; TNBC, triple‐negative breast cancer.

Aside from active interventions of the antigen generation and presentation processes in cancer cells, advances in DDSs have enabled the passive epitope loading of cancer cells. An antibody‐mediated delivery of viral MHC‐I epitopes was recently designed to evoke the activation and proliferation of virus epitope‐specific CD8^+^ T cells and delay tumour growth in murine breast cancer model.^54^ Manually loaded viral antigens could avert the sophistication in utilising cancer neoantigens, conferring cancer cell‐specific attacking to ‘off‐the‐shelf’ virus epitope‐targeted CTLs.

#### 2.2.2 APM defects in cancer cells

APM is a rather complicated system that extends across multiple biological processes such as protein degradation and vesicle transport and is responsible for the processing, transportation and presentation of antigen epitopes. In cancer cells, APM can be perturbed owing to various abnormalities (Figure 2). In vitro and in vivo CRISPR screenings have identified crucial participants in APM deficiency such as the inhibitory molecule of IFN‐γ signalling PTPN2 and methylation writer of genes involved in MHC‐I antigen presentation pathway polycomb repressive complex 2 (PRC2), providing latent cancer immunotherapy targets as well as emphasising the regulatory complexity of APM.^55^ Strategies rectifying the APM through repurposed drugs and DDSs have been proposed.

IFN signalling can intensify the transcription of crucial ‘gears’ in the cellular APM (e.g., immunoproteasome components and MHC molecules) and is a well‐dissected regulator of the APM. However, cancer cells can impede their IFN signalling through downregulating JAK1 and IFNGR1 or other mechanisms.^56^ Oncolytic virotherapy has been proven to harbour immunomodulating functions in addition to direct cytolytic effects. Yet, few studies shed light on their immunostimulatory effects on cancer cells except ICD. Intratumoural administration of live rotavirus vaccines revealed elevated expression of type I IFN pathway members in cancer cells. When rotaviruses were inactivated, the actuated IFN signalling was still guaranteed regardless of the lost ICD‐inducing capability, which implied the latent function of oncolytic virotherapy to promote antigen presentation.^57^ Epigenetic mechanisms are also involved in the aberrant APM function of cancer cells. DNA hypermethylation and H3K27 in APM‐related genic regions, especially those encoding the subunits of MHC molecules, can dampen the antigen presentation of cancer cells.^58^ DNMT1 inhibitors and HDAC1 inhibitors can induce the expression of APM‐related genes, highlighting the immunomodulatory capacity of epigenetic regulators.^59^ Similarly, after locking down ligand‐dependent corepressor (LCOR) as a master regulator of cancer cell APM, extracellular vesicles derived from *LCOR* knock‐in HEK293T cells, which contained a large amount of *LCOR* mRNAs, were administered to preclinical breast cancer models to elevate the LCOR level in cancer cells.^60^ The restored LCOR expression led to increased immunoproteasome activity, elevated MHC expression and many other APM‐related benefits and consequently revived the cancer cell antigen presentation.

In addition to the comprehensive activation of related biological events, APM can be remedied by directly targeting a specific protein functioning in antigen processing. Atractylenolide I, derived from an ancient Chinese herb, can stabilise immunoproteasome components PSMD4 and PSMD7, guaranteeing the proteasome activity and consequently leading to boosted antigen processing function in a mouse colorectal cancer model.^61^ These findings could inspire the research community to strengthen the APM for more effective anticancer immune responses from multiple biological aspects.

### 2.3 Infiltration, maturation and antigen presentation of APCs

Desired anticancer adaptive immune responses rely on valid antigen presentation by APCs like conventional DC1. To accomplish the cross‐presentation, DCs undergo recruitment, antigen internalisation and processing, maturation and migration towards the tumour‐draining lymph nodes (TDLNs).^62^

Cancer cells can recruit DCs by releasing chemokines such as CCL4 and CCL5 during the activation of inflammatory signalling pathways under intrinsic stresses including DNA damage.^63^ Although specific components in cell lysates such as ATP could act as a ‘find‐me’ signal to attract DCs as well,^64^ limited studies focus on the specific recruitment of DCs probably due to the lack of rational agents that can be precisely delivered to tumour sites. To overcome this dilemma, a sustained ATP‐releasing poly(lactic‐co‐glycolic acid) (PLGA) microparticle was developed and administered in murine melanoma models.^65^ An increasing number of DCs was found infiltrated into tumour sites and matured in TDLNs, suggesting targeted ATP delivery as a novel measure to augment APC infiltration.

An alternative tack to recruit DCs is to promote the formation of the tertiary lymphoid structures (TLSs) in tumour sites. TLSs are lymphoid organs formed by well‐organised immune cells in non‐lymphoid tissues under chronic inflammation. TLSs harbour the potential to recruit immune cells and initiate tumour‐specific cellular and humoral immune responses.^66^ Through the administration of well‐designed allogeneic vector cells (HEK293) carrying NKT cell ligand and tumour antigen mRNAs, TLSs were formed with enriched CD11c^+^ DCs in mouse melanoma followed by elevated innate and adaptive immune responses.^67^ This result supports recent studies identifying that the density, distribution and function of TLSs were significantly correlated with survival and recurrence in several human cancers.^68^ Importantly, we should note the dual faces of TLSs in cancer treatment, as a few reports have linked TLSs to unfavoured behaviours of cancers including metastasis, which prompts us to further dissect the associations of the maturation and cell composition of TLSs and cancer immunology.^69^

Diversified solutions aiming at the activation and maturation of DCs have been proposed including immune adjuvant delivery. Recently, a self‐assembled nanoadjuvant, poly(*L*‐phenylalanine)‐*block*‐poly(*D*‐lysine), was synthesised based on the immunogenicity of *D*‐amino acids. After being complexed with ovalbumin, the nanovaccine effectively induced DC maturation and antigen presentation process.^70^ Albeit highly dependent on targeted delivery systems, repurposed drugs have also been demonstrated to enhance the function of DCs and are summarised in Table 1.

## 3 ANTICANCER IMMUNE RESPONSE OUTSIDE THE TUMOUR

**FIGURE 3:**
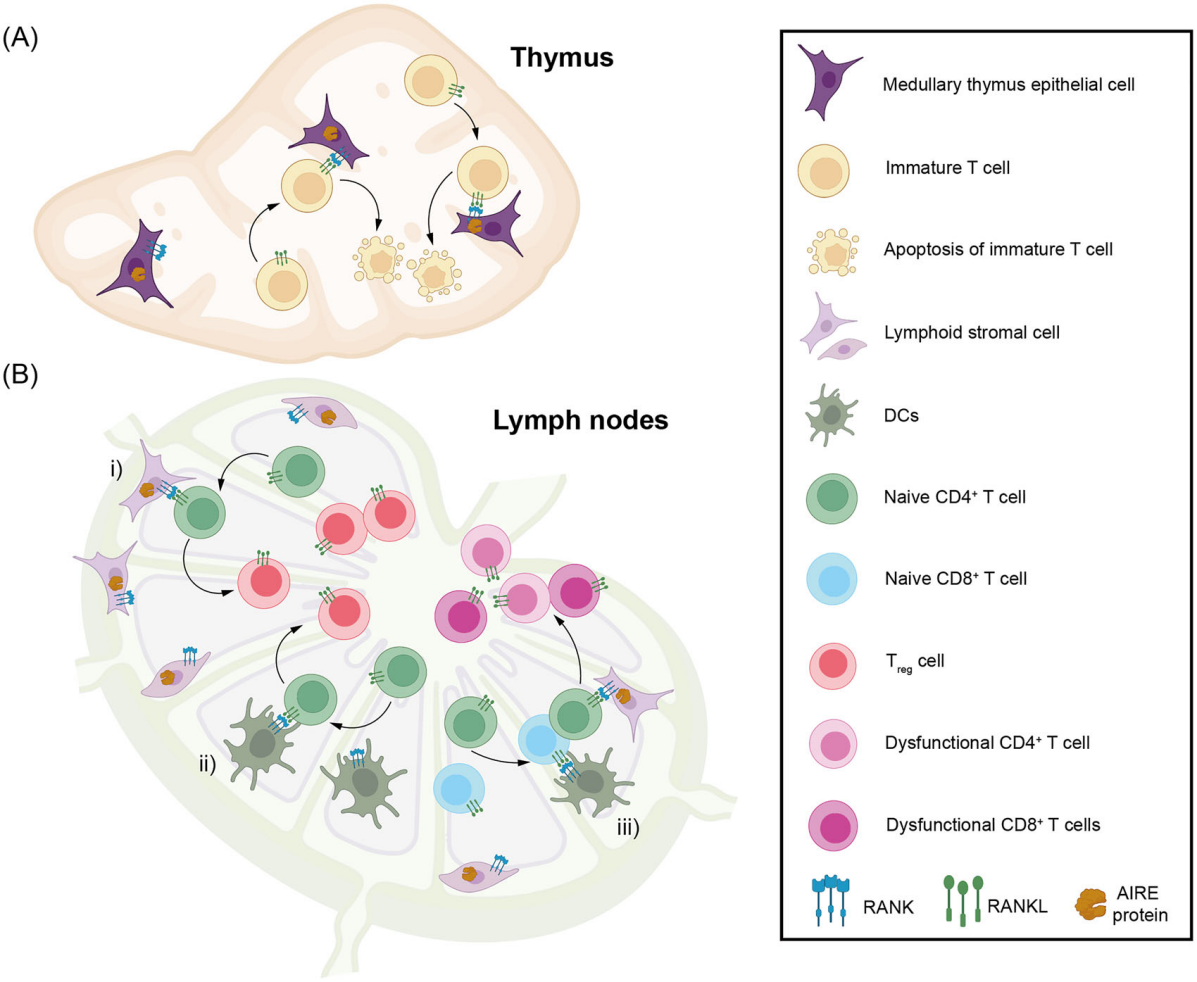
Receptor activator of NF‐κB (RANK/RANKL) signalling in central and peripheral tolerance. (A) The medullary epithelial cells (mTECs) in thymus expressing autoimmune regulator (AIRE) can express high level of self‐antigens. Interaction of immature T cells recognising self‐antigens with mTECs will lead to their apoptosis. During this procedure, interaction of RANK expressed by mTECs and RANKL expressed by immature T cells elevates the expression of AIRE in mTECs, thus leads to an enhanced negative selection in thymus. (B) Stromal cells in lymph nodes as well as DCs also express RANK. (i, ii) The RANK/RANKL signalling can result in the differentiation of naïve CD4^+^ T cells into T_reg_ cells after contact with lymphoid stromal cells or DCs. (iii) RANK/RANKL signalling can also lead to the anergy of naïve CD4^+^ and CD8^+^ T cells, mediating peripheral tolerance.

Cancer should be considered a systemic disease because it can exert negative influences on the whole immune system.^71^ Unfortunately, reversing the systemic immunosuppression in cancer patients remains challenging. Cytokine therapy was purposed to unleash the anticancer immune response systemically but received limited benefits, demonstrating that it may not be a one‐size‐fits‐all tactic.^72^ Repurposed drugs (Table 1) and DDSs (Table 2) have been developed to tackle this critical issue from various aspects (Figure 3).

### 3.1 Activation and proliferation of immune effector cells

#### 3.1.1 Immune tolerance in cancer immunology

Immune tolerance is an important regulatory mechanism taking place in both central and peripheral lymphoid organs, which removes self‐antigen reactive immune cells at early stages of development or mediates their anergy or deletion.^73^, ^74^ Therefore, it plays a key role in avoiding autoimmune disease and keeping the immune homeostasis. The establishment of immune tolerance largely depends on stromal cells expressing ubiquitous self‐antigens in the thymus (e.g., the medullary thymic epithelial cells) as well as APCs and regulatory immune cells in the periphery (e.g., immature DCs).^75^, ^76^ Tumours can highly express antigens that are also expressed in normal tissue at relatively low levels, termed tumour‐associated antigens (e.g., carcinoembryonic antigen). Tolerance to this type of antigen is responsible for the suppressed anticancer immune response.

Autoimmune regulator (AIRE) plays a pivotal role in immune tolerance for their transcriptional regulation of various tissue‐specific antigens both in the thymus and peripheral lymphoid organs.^77^ AIRE‐related tumour‐associated antigen‐reactive clone depletions of both CD4^+^ and CD8^+^ T cells can culminate in suppressed immune responses.^78^ The receptor activator of nuclear factor kappa‐B (NF‐κB) (RANK) is mainly expressed in stromal and myeloid cells of lymphoid organs, and its ligand RANKL is mainly expressed on effecter immune cells including CD8^+^ T cells. The interaction of RANK with RANKL facilitates the AIRE expression and function in Aire^+^ immune tolerance‐mediating cells.^79^ Thus, blocking RANK/RANKL signalling could be a putative therapeutic in augmenting anticancer immune response (Figure 3). Denosumab is an anti‐RANKL mAb initially developed to block the osteoclast‐associated RANK/RANKL signalling to prevent bone loss in osteoporosis and pathologic osteolysis in tumour bone metastasis.^80^ This drug was demonstrated to effectively deplete the Aire^+^ cells in lymphoid organs, rescuing tumour antigen‐reactive thymic CD4^+^ T cells in melanoma models. Repurposing denosumab in primary cancer treatment revealed its favourable impacts on anticancer immune responses, including suppression of the differentiation into regulatory T cells (T_reg_), increasing tumour‐infiltrating CD8^+^ T cells, abolishing the function of MDSCs and so forth.^79^ Clinical trials evaluating denosumab for cancer treatment are emerging, and the negative results from trials of treating early‐stage breast cancer and advanced non‐small cell lung carcinoma illustrated the fact that seeking the outlets of anti‐RANKL therapy in cancer immunotherapies was still crucial.^81^ Coupled with the success of PD‐1 blockade in *Aire*‐deficient colon cancer models, these discoveries might further render immune tolerance abrogation accessible in cancer immunotherapy.^82^

#### 3.1.2 Immune effector cells or immunosuppressive cells

Chronic stress is prevalent in patients with cancer, which is associated with immunosuppression, hastened tumour progression and therapeutic resistance.^83^ The master regulator of stress responses, adrenergic signalling, is always hyperactivated in such conditions. β‐adrenergic receptors expressed on immune cells can perturb the functions of effector cells.^84^ β‐adrenergic receptor blocker was found to introduce a significant decline of MDSC in the spleen and peripheral blood in tumour‐bearing mouse models, resulting in effective tumour control.^85^ The above findings provide us with evidence for repurposing β‐blockers as an immune booster in cancer therapy. Nonetheless, aerobic exercise‐activated β‐adrenergic signalling can increase the circulating T_eff_ cells in murine pancreatic cancer models, which urged a comprehensive interpretation of the interaction between the neuroendocrine system and anticancer immune responses.^86^

The immunostimulatory effects of TKIs were not restricted to cancer cell‐based mechanisms. Ibrutinib, a Bruton's TKI, can remarkably increase peripheral CD4^+^ and CD8^+^ T cells in leukaemia patients through the off‐target inhibition of IL‐2‐inducible T cell kinase (ITK) in T cells.^87^ Clinical trials based on the ITK‐inhibition characteristic of ibrutinib have been carried out in several solid tumours (Table 3). Other drugs such as anti‐inflammatory drugs were also identified to harbour repurposing potential as systemic immunomodulators in cancer treatment (Table 1).

Various DDSs have been developed with surface modifications to directly deliver stimulatory substances to circulating immune cells, for instance, modified by antibody targeting T cells.^88^ A gold nanoparticle loaded with a small molecule transforming growth factor beta (TGF‐β) inhibitor and conjugated with anti‐CD8 antibodies resulted in a remarkable abrogation of TGF‐β signalling in circulating CD8^+^ T cells.^89^ Another interesting example was a tissue factor‐based fusion protein complex carrying the extracellular domain of TGF‐β receptor II, IL‐15 and IL‐15 receptor α chain.^90^ The IL‐15‐IL‐15 receptor α chain can augment the activation of circulating NK cells and CD8^+^ T cells while the immunosuppressive actions of TGF‐β were partially diminished.

### 3.2 Sustainable immune response against tumour (re)challenge

One of the major challenges facing immunotherapy against cancer is the difficulty in eliciting a sustained immune response against cancer cells, which may trigger tumour recurrence or/and metastasis due to minimal residual diseases or the existence of cancer cells bearing drug‐resistant progenitor phenotypes.^91^ Memory T cells form the line of defence against tumour rechallenge. Differentiation and maintenance of memory T cell lineages are finely regulated by a complex interplay of transcriptomic and epigenetic factors, and some of them such as the well‐known TCF1 and newly identified ZNF683 have emerged as subgroup markers of memory T cells.^92^ Similar to chronic virus infection, sustained exposure to tumour antigen is associated with loss of TCF1 expression in T_eff_ cells, and TCF1 is a critical memory T cell characteristic known as the maintainer of the memory phenotype.^93^ Numerous evidence has underscored the importance of the memory T cell pool in combating cancer rechallenge.

Pretreatment with dacarbazine, a chemotherapeutic drug, can stimulate tumour‐specific effector memory T (T_em_) cells and enlarge the T cell receptor (TCR) repository in the peripheral blood of murine melanoma model administered with peptide vaccines.^94^ The Akt inhibitor (Akt inhibitor VIII) could generate tumour‐specific CD62^+^ T cells with memory phenotype in adoptive cell transfer using tumour‐infiltrating lymphocytes, which in return protected the mouse from tumour cell rechallenges.^95^ To repurpose these agents as memory phenotype‐inducers is fascinating, which can presumably expand their indications in cancer treatment.

Despite being phenotypically summarised, the cellular origin and differentiation mode of cancer‐associated memory T cell repository is not fully understood, and several hypotheses trying to explain this enigma have been proposed.^96^ Dissecting this cell lineage can provide more insights into establishing an immunological barrier against cancer rechallenge.

### 3.3 T helper (Th) 1:2 cell ratio

Surgery is widely used for cancer treatments, and anaesthesia is needed throughout the operation. Accumulating evidence suggests that the selection of anaesthetic drugs, approaches and time can exert diverse repercussions on the immune system. For instance, intravenous injection of dezocine can elevate the Th1/Th2 cell ratio after breast cancer surgery.^97^ The ratio of Th1/Th2 cells is associated with tumour growth, metastasis and survival, and the shift to Th1 cells tends to usher in a more desired immune response against cancer.^98^ These findings suggest an underappreciated role of anaesthesia in cancer immune response, which warrants further investigation.

### 3.4 DDSs targeting lymphoid tissues

Peripheral lymphoid organs, or secondary lymphoid organs (SLO), are considered the primary locations where the adaptive anticancer immune response is generated. Among SLOs, TDLNs undertake the most essential functions comprising antigen presentation and differentiation of effector cells.^99^ DDSs have been developed based on the lymphoid organ structure and cell types using a variety of strategies for the effective delivery of immunostimulatory agents (e.g., ICI mAbs or cancer vaccines) into TDLNs.

An intradermally administered, multistage‐draining lymph node (dLN)‐targeted DDSs with a programmable target visiting feature was designed.^22^ The release time of drugs could be controlled by the addition of different oxanorbornadienen linker substituents to this DDS, and the loaded CpG could be uptaken by cortex‐resident B cells and T cells, leading to their proliferation. There were other reported strategies in designing and meliorating lymph node‐targeted DDSs, such as altering the material compositions (e.g., zwitterionic poly(carboxy betaine) polymer)^100^ or exploiting ligands for specific binding (e.g., using antibodies to bind lymphatic endothelial cells).^101^ These DDSs are promising in the targeted delivery of immunostimulatory agents into TDLNs.

Previously, we have introduced strategies for promoting the formation of TLSs.^67^ Because of the shared structure of TLSs with lymph nodes such as the draining system and the high endothelial venules, it is anticipated that similar techniques may be applied to target TLSs for immunostimulation. Nonetheless, lymphatic metastasis is a common phenomenon. After being colonised by metastatic cancer cells, the microenvironment of dLNs is altered, and various immunosuppressive events including chronic IFN exposure and elevated PD‐L1 expression have been identified.^102^ Thus, before applying lymphoid tissue‐target drug delivery clinically, it is better to further holistically scrutinise the role of TDLNs and TLSs in cancer immunotherapies.

## 4 INFILTRATION OF EFFECTOR CELLS

**FIGURE 4:**
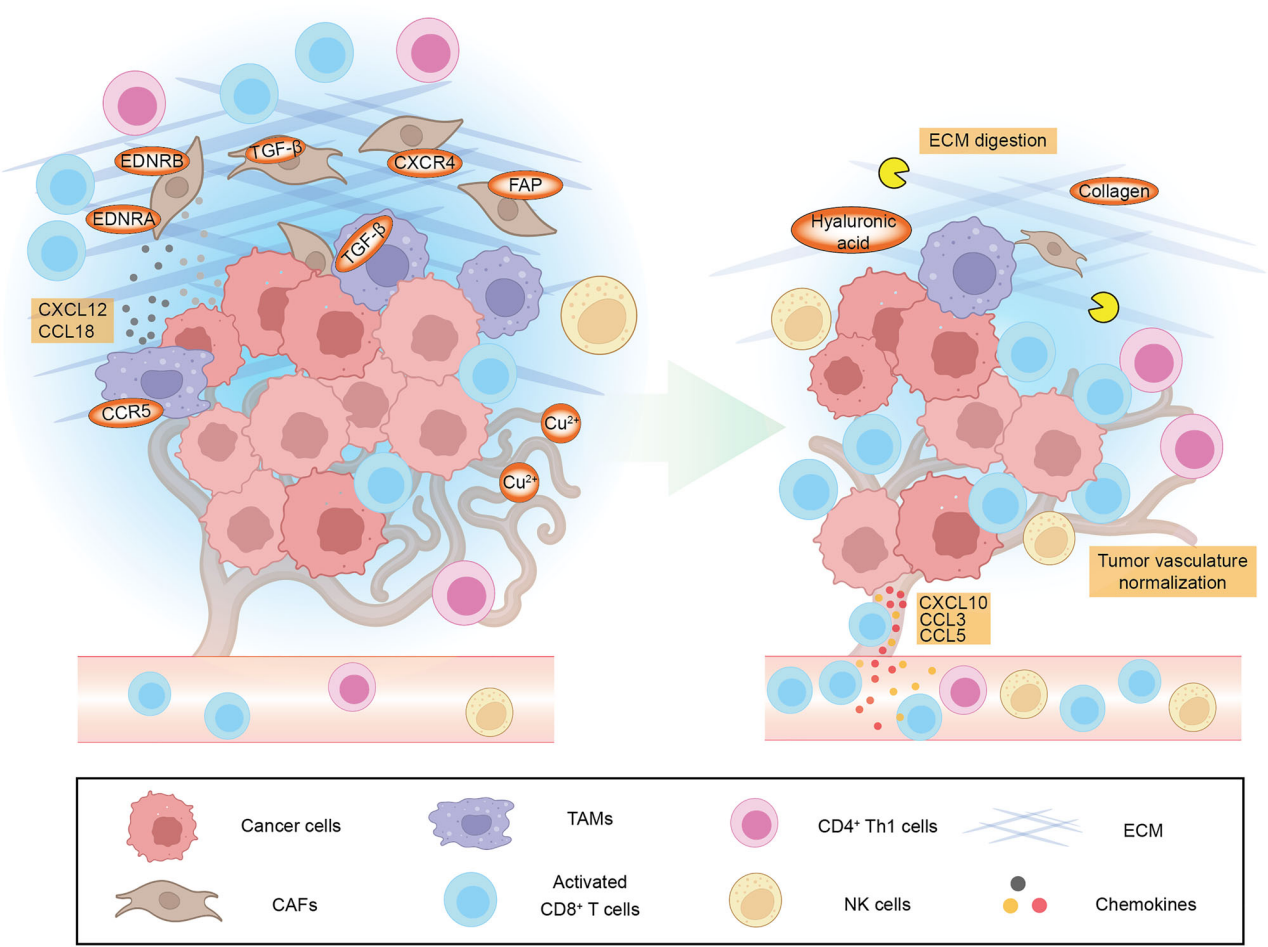
Alterations of TME components before and after repurposed drugs and DDSs treatment. Cancer‐associated fibroblasts (CAFs) can synthesise abnormal extracellular matrix (ECM) components, resulting in attenuated infiltration of effective immune cells. Cytokines secreted by tumour‐associated macrophages (TAMs) such as CCL18 and CXCL12 can recruit and enhance the function of CAFs. Blockade of these cytokine signallings through antagonising the corresponding receptors including CCR5 in TAMs and CXCR4 in CAFs suppresses their ECM synthesis by CAFs. Other CAF function‐related signallings such endothelin signalling and TGF‐β signalling, can be inhibited through blocking endothelin receptors (EDNRA and EDNRB) as well as TGF‐β. As a marker of fibroblast, fibroblast activation protein (FAP)‐targeted antibody can lead to the deletion of CAFs directly. ECM digestion of hyaluronic acid and collagen directly undermines the infiltration barriers, leading to elevated cytotoxic T lymphocytes and natural killer (NK) cells in TME. Meanwhile, the abnormal vasculature in tumours also hampers the infiltration of immune cells. Vessel normalisation substances such as copper chelators sponging Cu^2+^ in TME can also facilitate the infiltration of effective immune cells. These therapeutics could lead to the elevated numbers of infiltrated effective immune cells, alleviated ECM density and normalised vasculature.

‘Cold tumour’ is characterised by low infiltration levels of immune effector cells, especially cytotoxic CD8^+^ T cells, and its TME dominantly comprises immunosuppressive cell populations, such as T_reg_ cells, MDSCs, tumour‐associated macrophages (TAMs) and cancer‐associated fibroblasts (CAFs).^103^ The establishment of such an immune niche is associated with intricate factors, including tumour pathological types, mutations, antigenicity, metabolic features and so forth.^104^, ^105^ Currently, decoded mechanisms responsible for impeding effector cells at the invasion margin at least include (1) low antigen release and presentation levels; (2) insufficient activation of adaptive anticancer immune response; (3) immunosuppressive cytokines, metabolites or enzymes released by cells in TME; (4) extracellular matrix (ECM) components and solid stress; and (5) aberrantly organised vasculature. Stimulating immune infiltration is considered a valuable scenario for improving the anticancer immune response. Thus, a great demand for repurposed drugs and DDSs aiming at promoting immune infiltration is emerging (Tables 1 and 2). As immune infiltration can be rationally evoked by antigen presentation and immune activation we introduced previously, here we will focus on introducing the latter three mechanisms (Figure 4).

### 4.1 Effector cell‐recruiting cytokine signals

The vital biological processes of immune cells are regulated by the cytokine network. Some cytokines secreted by cells in TME, such as IL‐10, IL‐23, TGF‐β and CXCL12 have been well‐elucidated to be tumour‐promoting, hampering the anticancer immune responses in various ways.^106^ One way to break these suppressive signals is to amplify immunostimulatory signals in the cytokine network (Figure 4). Regorafenib, a multi‐target TKI approved for the second‐line treatment of several malignancies, was reported to enhance the infiltration of CD8^+^ T cells in hepatocellular carcinoma.^107^ An in‐depth exploration revealed that regorafenib upregulated CXCL10 expression in hepatocellular carcinoma cells through the indirect inhibition of STAT3, subsequently recruiting CXCR3^+^ lymphocytes, and this was independent of anti‐PD‐1 mAb administration. Therefore, the indirect inhibitory effect of multikinase inhibitors may again encourage the consideration of their repurposing in anticancer immunomodulation. Interestingly, analysis of solid tumour samples from patients receiving oncolytic virotherapy at single‐cell resolution revealed that virus replication was not restricted in tumour cells.^108^ Herpes simplex virus‐infected T cells and macrophages expressed significantly more CSF2 and IFN‐γ and could account in large part for elevated levels of infiltrated NK cells, monocytes and CTLs.

### 4.2 ECM modulation

ECM is of great importance for biological functions and microenvironment homeostasis in normal tissues. Whereas for cancer, many biochemical characteristics including macromolecular components, microtopography, density and porosity show significant differences, compared to normal tissues.^109^ As a consequence, numerous detrimental phenomena such as altered diffusion and perfusion of molecules, perturbed local regulation of growth factors and cytokines as well as abnormal cell adhesions and cell‐ECM interactions have been profiled and are tightly associated with cancer onset, progression, metastasis and immunosuppression.

#### 4.2.1 Suppression of the CAF recruitment and function

CAFs constitute the major mesenchymal cell population in TME, and the sources of CAFs comprise activated intratumour fibroblasts, migration (e.g., circulating fibrocytes), epithelial–mesenchymal transition and in situ transdifferentiation (e.g., smooth muscle cells), which harbour a high heterogeneity.^110^ Due to their non‐negligible features of synthesising and remodelling the tumour ECM together with many other immunosuppressive repercussions, CAFs are now approaching the forefront of cancer immunotherapy (Figure 4).

CXCR4 can be expressed by CAFs, and the CXCL12/CXCR4 axis was critical for the recruitment of immunosuppressive cells into TME.^111^ The selective CXCR4 antagonist, plerixafor, displayed decreased CAF recruitment in the TME followed by attenuated fibrosis and solid stress and increased CD8^+^ T cell infiltration.^112^ CCL18 secreted by TAMs can activate CAFs and promote their biosynthesis of ECM components. Blocking CCL18 production by TAMs through a CCR5 antagonist, maraviroc, remarkably circumvented the activation and matrix synthesis of CAFs.^113^ Despite the increasing number of early‐phase clinical trials focusing on cytokine‐based immunomodulating strategies in cancer treatment,^114^ some paradoxical effects (for instance, the aforementioned CCL5/CCR5 signalling participates in both NK cell and CAF recruitments) may imply that targeting the cytokine network for immunomodulation still requires ample investigations for understanding their biological functions and potential roles to serve as therapeutic targets. Besides recruitment, the function of CAFs is associated with signalling pathways such as osteopontin, phosphatidylinositol 3‐kinase (PI3K)/ activating serine/threonine kinase (AKT)/ mammalian target of rapamycin (mTOR) and IL‐1β signalling, providing CAF‐targeting therapeutic opportunities.^115^ Repurposed drugs targeting the TGF‐β signalling and endothelin signalling have been demonstrated to dampen the hyaluronan‐synthesising capacity of CAFs (Table 1).

Through single‐cell dissecting and validation,^116^ proteins such as fibroblast activation protein (FAP) and vimentin have been recognised as the markers of CAFs.^117^ Borrowing concepts from ICIs, mAb targeting FAP (sibrotuzumab) was developed but failed to elicit treatment response in clinical trials.^118^ To this end, improved strategies for targeting FAP have been reported, including CAR‐T therapy (NCT03932565). In terms of DDS, carriers with surface modification of FAP derived from tumour cells turned out to induce strong CTL responses against FAP^+^CAFs and modified TME.^119^ Due to the complex interactions of CAFs with immune cells, it may deliver further benefits by targeting CAFs beyond ECM normalisation.

#### 4.2.2 ECM digestion

Apart from CAF‐oriented strategies, ECM can be directly remodelled by local‐regional delivery of ECM component‐degrading enzymes, such as hyaluronidase and collagenase (Figure 4). Recently, an immune cocktail therapy consisting of doxorubicin, shRNAs against PD‐L1 and plasmid‐encoding hyaluronidase was proposed.^120^ Through poly(L‐glutamic acid)‐g‐methoxy PEG nanoparticle‐based DDS, successful expression of hyaluronidase in tumour cells was confirmed followed by an increase in CD8^+^ T cells due to the degradation of hyaluronic acid in ECM. An alternative scheme is to harness genetic engineering bacteria for hyaluronidase delivery.^121^ Collagenase can be similarly applied for ECM remodelling, but the delivery should also be highly targeted to avoid collagen digestion in normal tissues.

These results indicated that remodelling ECM has direct actions on immune infiltration. Accompanied by additional benefits such as elevated penetration of anticancer agents especially macromolecular ones,^122^, ^123^ ECM remodeling is apparently becoming a focused topic in cancer immunotherapy. Despite the improved overall response rate after the addition of hyaluronidase to nab‐paclitaxel/gemcitabine in treating hyaluronan‐high metastatic pancreatic adenocarcinoma, the combination of hyaluronidase with pembrolizumab failed to meet the primary endpoint (NCT02563548). Therefore, the research community is exploring more ECM remodelling‐oriented treatment strategies such as the MORPHEUS‐PDAC trial (atezolizumab + PEGylated human recombinant PH20 hyaluronidase vs. chemotherapy in metastatic pancreatic adenocarcinoma) as well as preclinical hyaluronidase‐encoding mRNA‐equipped CAR‐T therapy.^124^

### 4.3 Tumour vasculature normalisation

Cancer tissues can establish neovasculature through intricate means such as capitalising pro‐angiogenesis cytokines and vascular mimicry.^125^ These tumour vessels are characterised by disorganisation and hyperpermeability, tightly linking to a highly immunosuppressive TME and treatment resistance to chemo‐, radio‐ and immunotherapy. Hence, anti‐angiogenic therapy that blocks pro‐angiogenesis signallings such as vascular endothelial growth factor (VEGF)/ VEGF receptor (VEGFR) signalling pathway, is becoming a vital therapeutic option in various cancer types (Figure 4). For instance, atezolizumab plus bevacizumab performed better than sorafenib in the IMbrave150 trial and has been recommended as the first‐line treatment of advanced hepatocellular carcinoma, with another phase II/III clinical trial assessing the sintilimab plus bevacizumab biosimilar completed.^102^, ^126^

DDSs have already been investigated for the potential of anti‐angiogenic therapy against cancer. Tumour angiogenesis is a localised event that is intimately associated with the angiogenic signalling in TME. As such, DDSs may possess inherent advantages in anti‐angiogenic therapy owing to their tumour‐targeting capabilities, which was demonstrated by the greater therapeutic efficacy of bevacizumab delivered by alginate hydrogel.^127^ When administrating self‐assembling peptide amphiphile nanoparticles consisting of FSEC peptides and PD‐L1‐binding peptides ^D^PPA, the former peptides were released by the breakage of a legumain‐sensitive amino acid sequence from ^D^PPA peptides. They mediated the structural and functional normalisation of tumour vessels via inhibiting the binding of VEGF to its receptor on endothelial cells. An increase in tumour‐infiltrating NK cells and CD8+ T cells was then confirmed.^128^ As another example, heparin sulfatase 1‐expressing bacteria conjugated with NPs carrying doxorubicin can actively colonise tumour sites and dampen cancer angiogenesis and metastasis. Mechanistically, heparin sulfatase 1 can remove the sulphate of heparan sulfate proteoglycan core proteins to disturb its regulation on vascular development.^129^ Other pathways such as Smad2/3 and angiotensin signallings could also result in tumour angiogenesis,^130^ and strategies targeting these pathways have also been developed (Table 2).

Meanwhile, preclinical studies have earlier suggested a potential role for copper in thwarting angiogenesis.^131^ To this end, clinically approved copper chelators, penicillamine, trientine and tetrathiomolybdate were tested on murine mesothelioma models. An increase in CD4^+^ T cell infiltration, and reduced tumour vessel diameter and endothelial proliferation, followed by delayed tumour growth, were observed.^132^ However, clinical trials of systemic application of penicillamine as anti‐angiogenic therapy failed to improve the survival of patients with glioblastoma.^133^ In this setting, DDSs for targeted delivery of copper chelators were developed and superior vasculature normalisation effects were obtained, compared with systemic administration.^134^ Similarly, targeted delivery of nitric oxide (NO) into TME can also lead to vasculature normalisation and ECM degradation,^135^ owing to the activation of matrix metalloproteinases and modulation of TAMs by NO.^136^

## 5 ENHANCEMENT OF IMMUNE‐KILLING EFFECTS

### 5.1 Alternatives for ICI besides mAbs

**FIGURE 5:**
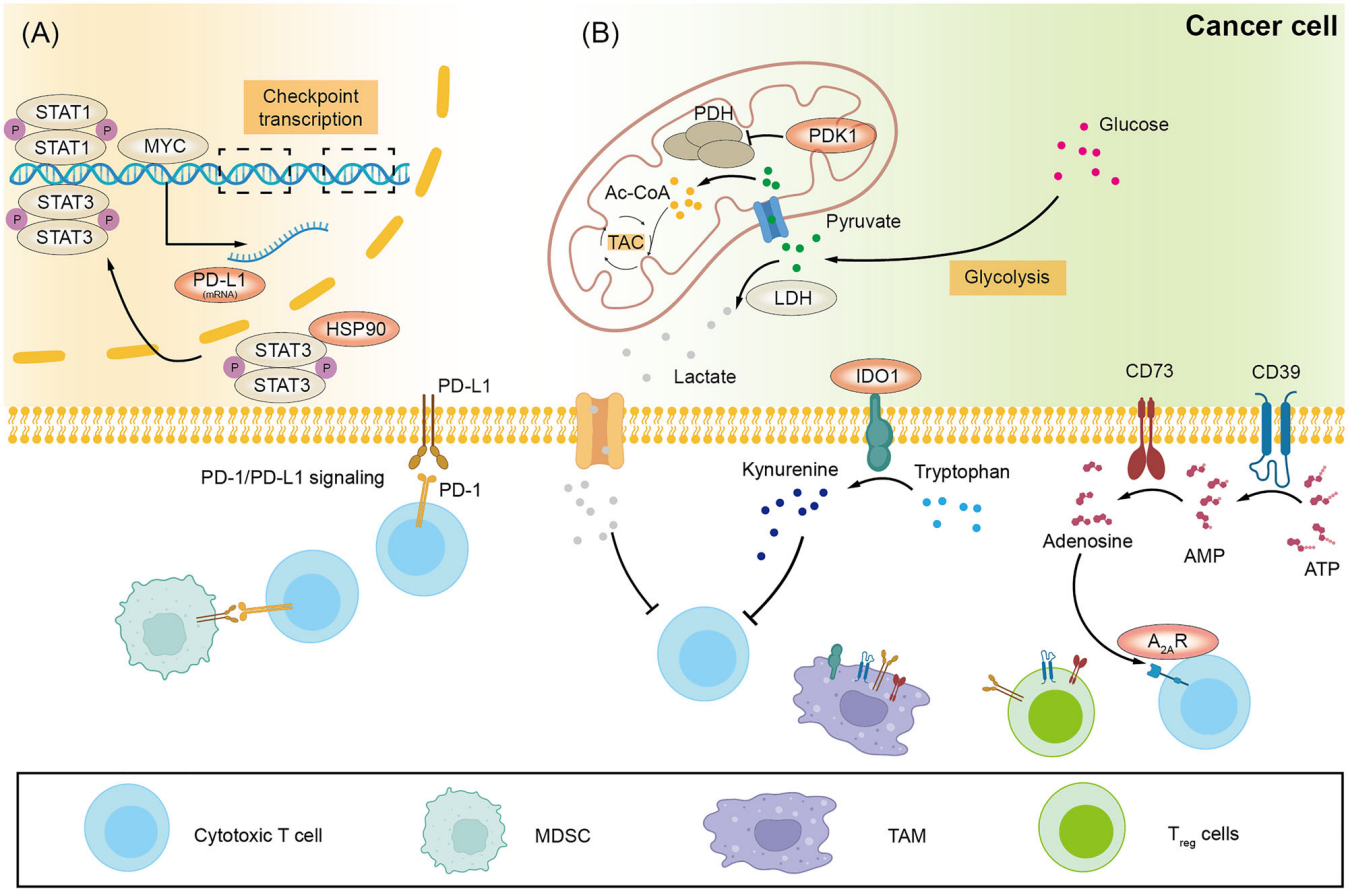
Therapeutic targets of repurposed drugs and DDSs for enhancing the tumour‐killing effects. (A) Tumour cells can express immune checkpoints such as PD‐L1 to suppress the function of effective immune cells. Through destabilising crucial transcriptional factors such STAT1, STAT3 and MYC by impairing the function of chaperones including HSP90, PD‐L1 expression can be abated at protein level. With the aids of DDSs, siRNAs targeting PD‐L1 can be effectively delivered into cancer cells, achieving checkpoint blockade at mRNA level. (B) Immunosuppressive metabolites, in particular, lactate, adenosine and kynurenine, can be generated by cells in TME including cancer cells, T_reg_ cells, TAMs and so forth via membrane enzymes including IDO1 and CD39‐CD73 axis. Through the inhibition of PDK1, the metabolic flux of glycolysis in cancer cells is intensified, subsequently leading to reduced lactate generation by LDH, its efflux into TME as well as the metabolic shift towards tricarboxylic acid cycle (TAC). Other repurposed drugs and DDSs could block IDO1 and CD39‐CD73 axis, diminishing kynurenine and adenosine levels, respectively, and these strategies could target myeloid‐derived suppressor cell (MDSC), TAMs and T_reg_ cells as well. AMP, adenosine monophosphate; ATP, adenosine triphosphate; PDH, pyruvate dehydrogenase; TAC, tricarboxylic acid cycle.

The approval of the anti‐CTLA‐4 mAb ipilimumab in 2013 for the treatment of advanced melanoma initiated the era of ICI in cancer treatment.^137^ Nowadays, immune checkpoints targeted by antibodies approved or in clinical trials include PD‐1 and PD‐L1, CTLA‐4, LAG‐3, TIGIT, TIM‐3 and VISTA.^138^, ^139^, ^140^, ^141^ Through a T cell‐membrane protein interactome, Siglec‐15 was unveiled to be a potential checkpoint in 2019, and mAb targeting Siglec‐15 is under investigation in clinical trials (NCT04699123). These ICI treatments are believed to reverse the exhaustion of effector cells by blocking immune checkpoint signalling in TME. But care should be taken because ample evidence suggests that the current systemic administration of ICI agents may give rise to immune‐related adverse events.^142^ Hence, strategies for more effectively targeting the checkpoint‐associated immunosuppressive signalling in the TME are emerging to avoid immune‐related adverse events. Here, we focus on those non‐mAb‐based ICI strategies (Figure 5).

Through linking the sequence of PD‐L1 binding peptides (PD‐L1pep1) and ferritin at oligonucleotide level, a PD‐L1pep1‐ferritin nanocage with the exposure of PD‐L1pep1 on the surface was expressed and extracted.^143^ PD‐L1pep1‐ferritin nanocages demonstrated high accumulation in tumour sites in vivo and effectively bound PD‐L1 in TME. In other cases, siRNAs and shRNAs can also be uneventfully transported to tumour sites and induced PD‐L1 knockdown through subtly designed DDSs.^144^ ICI can also be realised at the gene level through DDS (Table 2).

ICI therapies have been achieved through small molecule drugs (e.g., INCB086550 and ASC61), and some of them have been tested in clinical trials.^145^ During this process, repurposed drugs may also hold promise. Ganetespib is an HSP90 inhibitor originally envisaged for inducing cell cycle arrest and apoptosis of cancer cells.^146^ Recently, ganetespib was found to suppress the expression of several immune checkpoint molecules including PD‐L1, PD‐L2, B7‐H3 and B7‐H4 in cancer cells due to the destabilisation of related transcription factors such STAT3, which is a client protein of HSP90.^147^ Other repurposed drugs bearing the potential to mitigate the immune checkpoint‐mediated immunosuppression under systemic administration have been identified, and the off‐target inhibition of PD‐L1‐related transcription factors is their dominant mechanism (Table 1; Figure 5). Together, these strategies might offer alternatives beyond current antibody‐based ICI therapies.

### 5.2 Stimulation of tumour‐infiltrated effector cells

For several reasons, methods for direct activation and strengthening of the intratumour immune effect cells remain limited. An intuitive example is the adverse event of systemic cytokine or chemokine therapy.^106^ Nevertheless, the exploration of novel tactics to circumvent these limits continues.

Activated TCR signalling is a hallmark of tumour‐responsive T cells. Through exploiting the increased reduction potential of T cell surface after antigen recognition, a protein nanogels loading IL‐15 super‐agonist were designed and cross‐linked by a reduction‐sensitive linker.^148^ They competently reached the surface of intratumoural TCR‐activated T cells and elevated the number and cytotoxicity of intratumoural CD8^+^ T cells and NK cells. More importantly, such expansion and function enhancement were almost entirely absent in the circulating T cell population, restoring the safety of this activated T cell‐specific cytokine‐load DDS.

Steroid hormones have already been decoded to have drastic effects on the immune system, and the most well‐known one is the comprehensive immunosuppression brought by glucocorticoids. Through nuclear hormone receptor‐dependent or independent pathways, the function and fate of T cells can also be profoundly affected by steroid hormones.^149^ Ospemifene is a non‐hormone estrogen receptor modulator developed for menopause‐associated dyspareunia. In vivo experiments suggested that ospemifene could boost the production of Th1 cytokines such as IFN‐γ and IL‐2 in T_eff_ cells.^150^ However, during the repurposing of steroid hormones for cancer immunotherapy, their drug dosage and biphasic effects should be noted.^151^ Other drugs such as propranolol and vitamin C could also lead to an elevated function of tumour‐infiltrating cytotoxic cells reflected by increased expression of several effector cytokines (Table 1).

Owing to the retention in cancer and the initiation of the anticancer immune response through both cytolytic substances and immune cell recruitments, T_rm_ cells, characterised by high expressions of CD103 and CD49a,^152^ have attracted more attention among T cell subpopulations. Results from recent studies have suggested the key position of T_rm_ cells as early responders in neoadjuvant immunotherapy, further highlighting the unique role of the T_rm_ subgroup in anticancer immunity.^153^ Accumulating evidence has suggested that their source of energy is fatty acid‐dependent,^154^ and the maintenance of T_rm_ phenotype relies on epigenetic regulators including HDAC11 and DNMT3.^155^ The discovery of these possibly druggable targets could motivate the exploration of additional approaches for targeting the ‘spark’ of immune response in tumours.

### 5.3 Suppression of immunosuppressive cells in TME

TAMs represent one of the most studied immunosuppressive cells in TME. Their influences on anticancer immune response are multifaced, involving secreting immunosuppressive cytokines (e.g., IL‐10), T_reg_ recruitment, ECM remodelling directly or through CAFs and so forth.^156^ Therefore, the intervention of TAMs has become an essential topic in improving immune‐mediated tumour control, and the re‐education of TAMs towards a pro‐inflammatory M1‐like phenotype shows significant promise.

While some anticancer agents have been reported to serve as an immunomodulator to regulate macrophage polarisation in TME,^157^ there exist drugs that were not designed for tumour treatment but harbour TAM modulatory effects. A commonly used psychiatric drug, monoamine oxidase A inhibitor, was found to constrain the immunosuppressive polarisation of TAMs by blocking ROS‐induced activation of the JAK‐STAT6 pathway.^158^ Clinical data also supported monoamine oxidase A levels in patients after receiving PD‐1 or PD‐L1 mAbs treatments as a biomarker for worse clinical outcomes.^159^ The clinically approved perhexiline, which suppresses fatty acid transportation, can also prevent macrophages from acquiring M2 phenotype.^160^ Together with several repurposed drugs we summarised before, these results indicated the multifaced potential of repurposing drugs to modulate TAM polarisation and function (Table 1).

As a kind of phagocyte, TAMs are relatively apt to be targeted with DDSs for their phagocyting aptitudes as well as abundantly expressed innate‐immune‐related receptors. Nitrogen‐containing bisphosphonates such as zoledronic acid can modulate the function of TAMs and have long been selected as cargos for TAM‐targeting DDSs.^161^ To enhance the TAM‐targeting ability of zoledronic acid liposomes, sialic acid modification was incorporated and M2‐like TAMs were more efficiently killed or re‐educated to an M1‐like phenotype.^162^ The zoledronic acid‐loaded DDSs can be coupled with photosensitisers and immunomodulatory drugs to further exert holistic effects in terms of immunotherapy.^163^ DDSs carrying chloroquine reprogrammed the metabolic flux of TAMs into an M1‐like glycolysis‐dominant phenotype,^164^ and nanoparticles like Gd‐metallofullerenol and iron oxide were even capable of directly stimulating TAMs towards M1‐like polarisation without cargos.^165^ In addition to the aforementioned DDSs, there are many ingeniously designed ones targeting TAMs that have demonstrated effective TAM‐based anticancer effects in preclinical models, which were summarised in Table 2.

Previously, the goal of selectively targeting TME‐resident T_reg_ cells was met through mAbs against the glucocorticoid‐induced tumour necrosis factor receptor‐related receptor, which is constitutively expressed on the T_reg_ cell membrane.^166^ After this proof‐of‐concept, increasing strategies utilising T_reg_‐inhibiting agents for cancer immunotherapy emerged. Neuropilin‐1 (NRP1) is another feature protein expressed by T_reg_ cells, and surface NRP1 level was linked to an increasing number of intratumoural T_reg_ cells and reduced survival in cancer patients.^167^ A tLyp1 peptide‐conjugated hybrid nanoparticle was synthesised to deliver imatinib to target T_reg_ cells in TME.^168^ Passive accumulation in TME and precise binding of tLyp1 to NRP1 synergistically delivered imatinib into tumour‐infiltrating T_reg_ cells, and their immunosuppressive properties were thwarted by inhibiting the phosphorylation of STAT3 and STAT5. In addition to targeted strategies, repurposed drugs were also proven to dampen the function of T_reg_ cells in TME (Table 1).

MDSCs were not as easy as TAMs and T_reg_ cells to be directly targeted due to less specific surface markers and weaker phagocytosis. Despite such limitations, drugs with repurposing capacity to inhibit MDSCs in TME have been identified such as ibrutinib and propranolol (Table 1). DDSs can also reduce the infiltrated number of MDSCs through targeted delivery of TGF‐β inhibitor into TME.^169^ Nevertheless, more efforts are needed to modulate the intratumoural MDSCs for augmented tumour‐killing effects.

### 5.4 Tumour immunometabolism

Tumour harbours a massively altered metabolic landscape, which covers a broad spectrum of various cellular metabolic pathways.^170^ Notably, cancer cells can dampen the functions of immune effector cells, especially T_eff_ cells, through nutrient competition, hypoxia exposure and immunosuppressive metabolites and so forth.^171^ Therefore, targeting the immunometabolic interplay in TME may contribute to an elevated anticancer immune response (Figure 5). The hypoxic microenvironment together with many other mechanisms will lead to a glycolytic dominant metabolic milieu in tumour, which significantly elevates the lactate concentration in TME. Although numerous studies have illustrated the immunosuppressive effects of lactate, few breakthroughs in treatments targeting lactate have been achieved beyond experimental conditions because the glycolysis pathway is a pervasive and constitutive metabolic pathway.^172^ Through delivering the prodrug of dichloroacetate, a PDK1 inhibitor, into tumour mitochondria using modified PEG‐PLGA nanoparticles, the production and efflux of lactate into TME were remarkably inhibited in murine colorectal cancer models.^173^

In addition to the direct release of lactate, cells in TME can convert small molecules into immunosuppressive metabolites through membrane proteins. Kynurenine is such an immunosuppressive metabolite generated by indoleamine 2,3–dioxygenase 1 (IDO1).^174^ Several small molecule IDO1 inhibitors have been developed and investigated. Still, the phase III clinical trial of IDO1 inhibitor plus anti‐PD‐1 mAb in advanced melanoma failed to improve overall survival, compared with anti‐PD‐1 monotherapy, which implied the requirement for further improving IDO1 inhibition strategies.^175^ To this end, a complex DDS was designed to deliver IDO1 inhibitor (NLG919) into tumour sites, and the intratumour kynurenine concentration was dramatically reduced, with increased proportions of CD8^+^ T cells, T_em_ and DCs.^176^ Adenosine is another immunosuppressive metabolite derived from the sequential degradation of AMP by CD39 and CD73.^177^ Superparamagnetic iron oxide nanoparticles delivering siRNAs targeting adenosine receptors were applied to protect T_eff_ cells from adenosine in TME.^178^ Notably, in a phase I clinical trial of the small molecule A_2A_ receptor antagonist, ciforadenant, in treating refractory renal cell carcinoma, a relatively high incidence of fatigue (13 out of 33 patients, 39.4%) was reported.^179^ It might be due to the widespread distribution of adenosine receptors in the human body, which further underscores the necessity of targeted delivery when engaging adenosine‐focused therapeutics in cancer treatments. As we delve deeper towards the immunometabolic landscape, more targetable molecules associated with immunosuppressive metabolites such as heme oxygenase‐1 and ornithine decarboxylase have been discovered,^180^ which will support the subsequent exploration of repurposed drugs and DDSs (Tables 1 and 2).

### 5.5 Tumour inflammatory microenvironment

Unlike the previously introduced cytokine‐ or chemokine‐oriented strategies that usually focused on a specific cell population, the repercussion of dysregulated tumour inflammation microenvironment undoubtedly requires more complex modulations on the cytokine network.^181^ In the murine adult T cell lymphoma model, which is characterised by a constitutively activated NF‐κB signalling, tumour growth was successfully dampened through the use of a peptide‐modified self‐assembling RNA polyplex DDS loaded with siNF‐κB.^182^ However, in many other solid tumours, the benefits of inhibiting key inflammation regulators such as NF‐κB remain doubtful and require further explorations. Instead, there are strategies focusing on the downstream inflammatory regulators. For instance, previous evidence has suggested cyclooxygenase–2 (COX‐2) in TME as an underlying pro‐tumour factor. The selective COX‐2 inhibitor celecoxib can stimulate the anticancer immune responses by elevating the release of damage‐associated molecular patterns and reducing the PD‐L1 level of cancer cells.^183^, ^184^ Clinical trials of these anti‐inflammation‐targeted strategies in combination with immunotherapies were carried out to evaluate their effects on anticancer immune response (Table 3).

## 6 CONCLUSION AND FUTURE PERSPECTIVES

In this review, we introduced various kinds of drugs that may have the potential for repurposing as anticancer immune stimulators. A few mechanisms by which chemotherapeutic agents may augment anticancer immune responses through their immunomodulatory properties, including inducing ICD and hormesis effects, have been elucidated.^185^, ^186^ These understandings might push forward their repurposing as immunomodulators, especially in combination with other anticancer therapeutics, instead of being cytotoxic anticancer agents (Table 3). However, pharmaceuticals such as COX‐2 inhibitors and metformin possess intricate immune‐related anticancer mechanisms that span almost all stages of the anticancer immune response (Table 3). Recent accounts of unsuccessful clinical trials assessing these drugs may indicate the lack of a more comprehensive understanding of their immunomodulatory actions.^187^ Additionally, drugs provisionally considered to have shared mechanisms may eventually exert different effects on the anticancer immune response.^150^ Hence, a clear interpretation of mechanisms paves the way for successful repurposing, and our comprehension of drug repurposing should not be paused at phenomenon levels. In parallel, it is essential to record and evaluate the long‐term toxicity of repurposed drugs, as their altered usage (e.g., temporary use towards long‐term administration) may elicit unforeseen adverse reactions.

We also introduced various DDSs bearing immunostimulatory properties, displaying their capacities for cancer immunotherapy. Here, we would summarise two major trends in the design of DDSs. First, there is an increasing emphasis on structurisation and modularisation. From one introduced example, probiotics with autolytic features were genetically engineered to express ICI nanobodies.^188^ The genetically encoded cargos could thus be substituted without altering the established luxI *φX173E* autolysis assembly to impart new functions to this bacteria‐based synchronised cycle‐relied DDS.^189^ The modularity design of DDSs enables large‐scale screening and validation of latent therapeutic agents. The other trend is of increasing sophistication, such as a two‐step or three‐step cascade release of cargos triggered by different stimuli instead of a one‐off release.^190^ In vitro strategies involving magnetic field guiding have also been incorporated in upgrading the targeting ability of established DDSs.^191^ These efforts can competently elevate the drug concentration in targeted sites or cells and further reduce the off‐target effects brought by DDSs. Nevertheless, during the development of DDSs, the type of drug carriers, the administration methods and some technique parameters (e.g., the pore size of nanoparticles) all need to be finely tuned to avoid some unanticipated features. For instance, PLGA and CaCO_3_ microparticles were shown to worsen some cancer‐associated complications (e.g., ascites) and induce accelerated tumour growth in syngeneic mouse ovarian cancer models.^192^

When developing repurposed drugs and DDS for enhancing anticancer immune response, we must consider the heterogeneity of cancers. Taking driver gene heterogeneity as an example, an increased level of macropinocytosis in KRAS mutant cancer cells was noted, compared with KRAS wild‐type ones. Drugs delivered by dextran achieved an enhanced entry in a murine KRAS mutant pancreatic cancer model.^193^ Similarly, *BRAF*‐mutant melanoma displayed a decrease in infiltrating CD8^+^ T cells and an intensified angiogenesis level,^194^ which may refine current therapeutics to target angiogenesis.^195^ Heterogeneity also exists across cancer types, metastatic and recurrent tumours,^196^ which elicit diverse effects on anticancer immune response and TME components. Thus, drug repurposing and DDS development should capture the opportunities provided by tumour heterogeneity and avoid a one‐size‐fits‐all line of investigation models.

Currently, cancer immunotherapy keeps advancing, and novel solutions such as structural improvement of CAR‐T cells and bispecific immune checkpoint antibodies have been continuously sprung up, representing the future direction of cancer medicine. Two bispecific antibodies targeting PD‐L1/4‐1BB and PD‐1/CTLA‐4 have been evaluated in early‐stage clinical trials and demonstrated concrete immune effects.^197^, ^198^ Co‐administration of other agents to further boost the therapeutic effects of immunotherapy is now prevailing across the preclinical and clinical investigations (Table 3). The mechanisms of agents composing the combination regimen should be complementary instead of antagonistic. Anti‐CTLA‐4 mAbs instead of anti‐PD‐1/PD‐L1 mAbs were selected to be co‐administered with imatinib in murine melanoma models to synergistically inhibit T_reg_ functions since anti‐CTLA‐4 mAbs could deplete T_reg_ cells while anti‐PD‐1 mAbs could possibly enhance their immunosuppressive effects.^168^, ^199^

In conclusion, founded on the ‘chain reaction’ of the anticancer immune response, we systemically summarised the emerging drugs harbouring the potential for being repurposed as immunostimulators in cancer treatments and DDSs capable of delivering agents that can boost the anticancer immune response. Several caveats must be considered in developing repurposed drugs and DDSs. With more repurposed drugs screened out and DDSs established, they will eventually take up residence in the future application of immunotherapy in the form of monotherapy or combination regimens.

## CONFLICT OF INTEREST STATEMENT

The authors declare no conflicts of interest.

## FUNDING INFORMATION

National Natural Science Foundation of China (Grant 82130077); Research Projects from the Science and Technology Commission of Shanghai Municipality (Grants 21JC1410100, 21JC1401200)
